# Untargeted metabolomics for uncovering plasma biological markers of wet age-related macular degeneration

**DOI:** 10.18632/aging.203006

**Published:** 2021-05-04

**Authors:** Yanhui Deng, Ping Shuai, Haixin Wang, Shanshan Zhang, Jie Li, Mingyan Du, Peirong Huang, Chao Qu, Lulin Huang

**Affiliations:** 1 The Key Laboratory for Human Disease Gene Study of Sichuan Province and the Center of Laboratory Medicine, Sichuan Provincial People’s Hospital, University of Electronic Science and Technology of China, Chengdu, Sichuan, China; 2 Research Unit for Blindness Prevention of Chinese Academy of Medical Sciences (2019RU026), Sichuan Academy of Medical Sciences, Chengdu, Sichuan, China; 3 Health Management Center and Physical Examination Center of Sichuan Provincial People's Hospital, School of Medicine, University of Electronic Science and Technology of China, Chengdu, Sichuan, China; 4 Department of Ophthalmology, Sichuan Provincial People’s Hospital, School of Medicine, University of Electronic Science and Technology of China, Chengdu, Sichuan, China; 5 Shanghai General Hospital, Shanghai, China

**Keywords:** wAMD, CNV, PCV, plasma, metabonomics

## Abstract

Wet age-related macular degeneration (wAMD) causes central vision loss and represents a major health problem in elderly people. Here we have used untargeted metabolomics using UHPLC-MS to profile plasma from 127 patients with wAMD (67 choroidal neovascularization (CNV) and 60 polypoidal choroidal vasculopathy (PCV)) and 50 controls. A total of 545 biochemicals were detected. Among them, 17 metabolites presented difference between patients with wAMD and controls. Most of them were oxidized lipids (N=6, 35.29%). Comparing to controls, 28 and 18 differential metabolites were identified in patients with CNV and PCV, respectively. Two metabolites, hyodeoxycholic acid and L-tryptophanamide, were differently distributed between PCV and CNV. We first investigated the genetic association with metabolites in wet AMD (*CFH* rs800292 and *HTRA1* rs10490924). We identified six differential metabolites between the GG and AA genotypes of *CFH* rs800292, five differential metabolites between the GG and AA genotypes of *HTRA1* rs10490924, and four differential metabolites between the GG and GA genotypes of rs10490924. We selected four metabolites (cyclamic acid, hyodeoxycholic acid, L-tryptophanamide and O-phosphorylethanolamine) for *in vitro* experiments. Among them, cyclamic acid reduced the activity, inhibited the proliferation, increased the apoptosis and necrosis in human retinal pigment epithelial cells (HRPECs). L-tryptophanamide affected the proliferation, apoptosis and necrosis in HRPECs, and promoted the tube formation and migration in primary human retinal endothelial cells (HRECs). Hyodeoxycholic acid and O-phosphorylethanolamine inhibited the tube formation and migration in HRECs. The results suggested that differential metabolites have certain effects on wAMD pathogenesis-related HRPECs and HRECs.

## INTRODUCTION

Age-related macular degeneration (AMD) is one of the leading causes of blindness worldwide. As the world population ages, the number of people with AMD is expected to increase to 288 million in 2040 [1]. AMD is a multiple factor disease. Age, hypertension [2, 3], atherosclerosis [4], diabetic retinopathy (DR) [5, 6], smoking [7] and heavy drinking [8, 9] all increase the risk of AMD. Genetic factors also greatly contribute to the occurrence of AMD [10]. *Complement factor H* (*CFH*) [11–13] and *high temperature requirement factor A1* (*HTRA1*) [14–16] are two major susceptibility genes for AMD. In addition, *complement factor B* (*CFB*) and *complement component 2* (*C2*) [17], *complement component 3* (*C3*) [18], *age-related maculopathy susceptibility 2* (*ARMS2*) [19], *apolipoprotein E (APOE)* [20, 21] and *FGD6* [22] also play an important role in the development of AMD.

There are two main types of AMD: dry (atrophic) AMD and wet (exudative) AMD (wAMD). Dry AMD shows geographic atrophy and no blood or serum leakage [23]. Wet AMD occurs in approximately 10-15% of people who develop AMD in Western populations and a higher proportion in Eastern populations. Wet AMD has the obvious symptoms of leakage and neovascularization. Although controversial, wet AMD can be divided into choroidal neovascularization (CNV) and polypoidal choroidal vasculopathy (PCV) [24]. The pathology of wet AMD progresses more quickly than the pathology of the dry form. Wet AMD causing significant deterioration to central vision within a short period of time. At present, the pathogenesis of wAMD is not very clear.

Metabolites are produced by the cumulative effect of the genome and its interaction with the environment. It is thought to be closely related to the phenotype of diseases, especially multifactorial diseases [25]. Metabolomics is a new omics approach after genomics and proteomics, which is mainly to conduct qualitative and quantitative analysis of all low molecular weight metabolites of a certain organism or cell in a specific physiological period to explore the relative relationship between metabolites and physiological and pathological changes. Metabolomics has made important achievements in the study of cardiovascular diseases [26], breast cancer [27, 28], Parkinson's disease [29] and diabetes [30]. Recently, researchers have also discovered the potential and versatility of metabolomics in the study of eye diseases [31–33]. For AMD, Lains et al. reported metabonomics research mainly based on white ethnicity and found that the glycerophospholipid pathway was significantly enriched [34–36].

It is well known that microorganisms are closely related to human diseases. A recent study [37] showed that microbial characteristics may play an important role in the diagnosis of cancer. Rob Knight's team found unique microbial signals in blood and tissue samples from most cancer patients. They also found that using only plasma-derived, cell-free microbial nucleic acids can distinguish between healthy, cancer-free individuals and samples from a variety of cancer patients.

In this study, we conducted plasma metabonomics research in Asian ethnicity-base on a Chinese population. The current study has three goals: (1) to characterize the plasma metabolomic profiles of patients with wAMD and to compare them with those of controls (including wAMD vs controls, CNV vs controls, PCV vs controls, CNV vs PCV); (2) to characterize the plasma differential metabolites of participants with different genotypes of major associated genes *CFH* rs800292 and *HTRA1* rs10490924; and (3) to identify specific metabolites of microorganisms in plasma of patients with wAMD. Finally, we aim to support the development of novel metabolic biomarkers for wAMD diagnosis and prognosis, as well as for drug development.

## RESULTS

### Study population

Participants in this study included 127 wet AMD patients (67 CNV, 60 PCV) and 50 healthy people. The demographic characteristics of the three groups of participants are shown in Supplementary File 1.

### Screening of metabolites with significant differences

**Figure 1:**
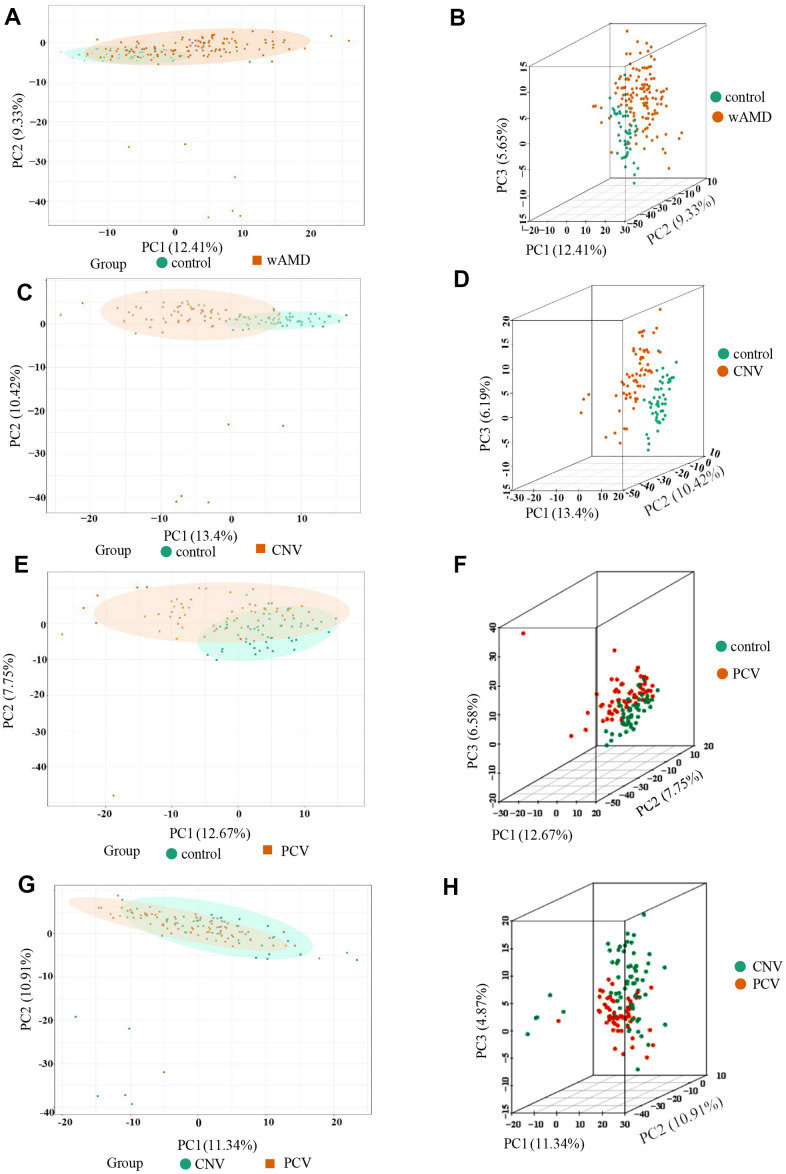
**PCA result of the wAMD group, PCV group, and CNV group.** (**A**, **C**, **E**, **G**) are the two-dimensional images of the PCA results of each group. (**B**, **D**, **F**, **H**) are the 3D images of the PCA results of each group. The X-axis represents the first principal component, the Y-axis represents the second, and the Z-axis represents the third. wAMD: represents the mixture of the CNV group and PCV group.

Three-dimensional principal component analysis (PCA) showed the trend of metabolites partially separated between groups, indicating differences among them [38]. The PCA results showed that the difference between the PCV group and CNV group was relatively small among all the comparison group, while the difference between the control group and CNV group was relatively large among all the comparison group (Figure 1).

Using the partial least squares discriminant analysis (PLS-DA) model, we calculated the comparison results between groups (Supplementary File 2). In wAMD compared with controls, 164 metabolites with VIP values >1 accounted for 30.10% of all metabolites detected. In CNV compared with controls, 171 metabolites had VIP values >1 (31.38%). In PCV compared with controls, 159 metabolites had VIP values >1 (29.18%). In CNV compared with PCV conditions, 145 metabolites had VIP values > 1 (26.61%). Then, according to the screening criteria for significantly differential metabolites discussed in the Methods section, totally 24 significantly differential metabolites were detected between disease conditions and controls. These metabolites include oxidized lipids (25.00%), benzene and its substituted derivatives (16.67%), nucleotide metabolism (12.50%) and amino acid metabolism (12.50%) (Table 1).

**Table 1:** Types of differential metabolites.

**Class**	**Compounds**	**Proportion (N=24)**
Alcohol	1-Aminopropan-2-ol	4.17%
Amino Acid metabolomics	L-Tryptophan; Trimethylamine N-Oxide; L-Alanyl-L-Lysine	12.50%
Bile Acids	Hyodeoxycholic Acid	4.17%
Benzene and substituted derivatives	2,6-Di-tert-butyl-4-methylphenol; 2-Methylbenzoic acid; 2,4-Dihydroxybenzoic Acid; 1,2,3-Trihydroxybenzene	16.67%
Benzoic Acid and its derivatives	2-Methoxybenzoic Acid	4.17%
Co Others Enzyme Factor and vitamin	Vitamin D3	4.17%
Lipids Others Phospholipid	O-Phosphorylethanolamine	4.17%
Nucleotide metabolomics	UDP-glucose; Phosphocholine; 1-Methylxanthine	12.50%
Organic Acid and its derivatives	1-Methyluric Acid; Carbamoyl phosphate	8.33%
Oxidized lipid	(±)4-HDHA; (±)12-HEPE; (±)12-HETE; 14(S)-HDHA; (±)9-HETE; 15-oxoETE	25.00%
Phenols and its derivatives	Hydroquinone	4.17%

### Metabolites with significant differences in wAMD vs controls

**Figure 2:**
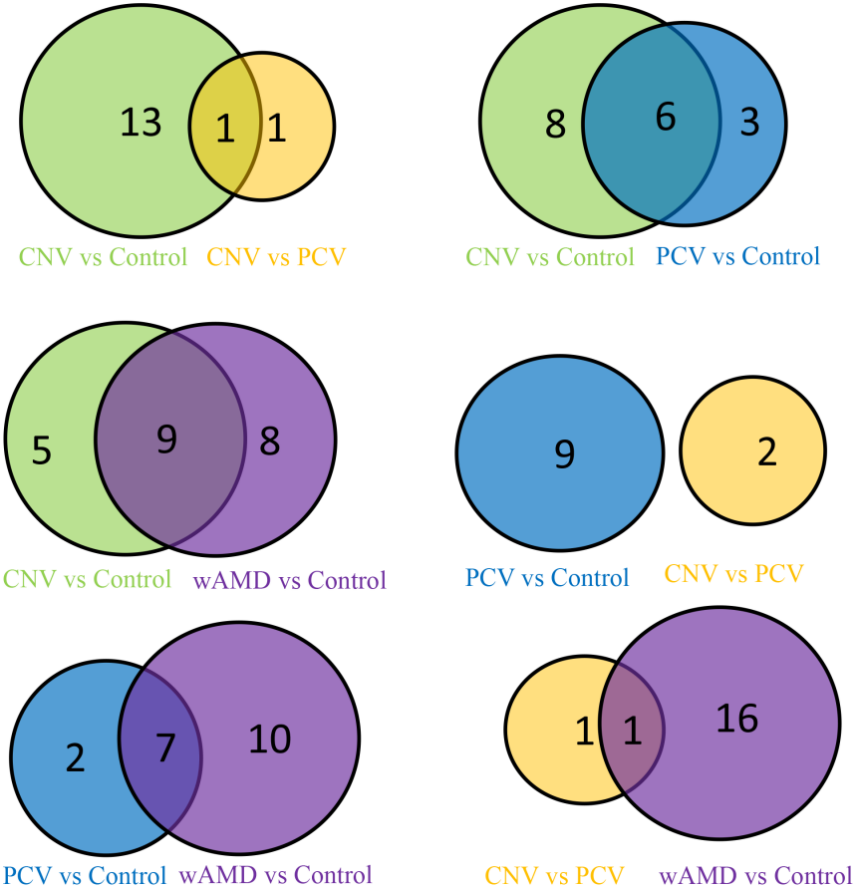
Venn map of the wAMD group, PCV group, and CNV group.

**Figure 3:**
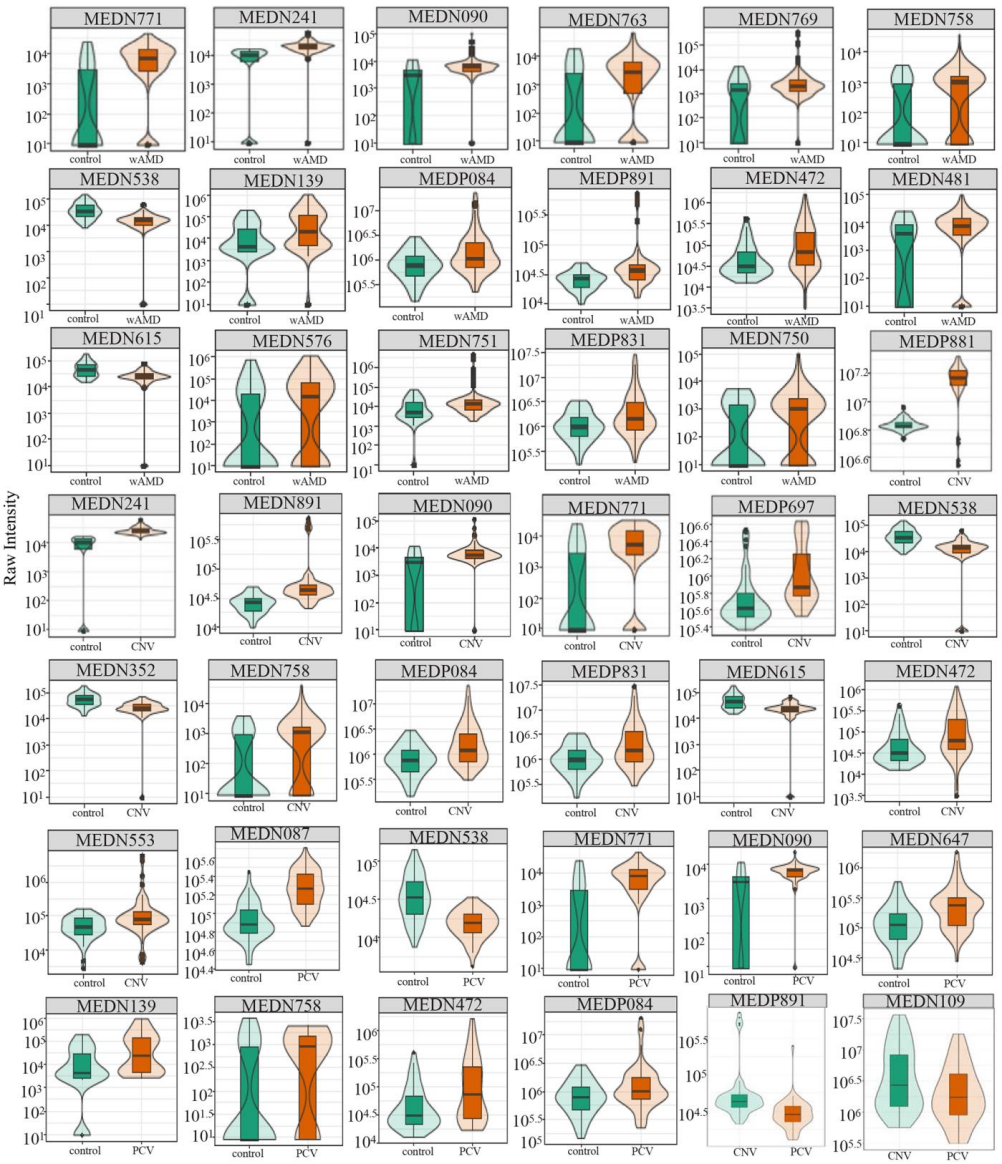
**Relative contents of differential metabolites among wAMD group, CNV group, and PCV group.** The box in the middle represents the quartile range, the thin black line extending from it represents the 95% confidence interval, the black horizontal line in the middle is the median, and the external shape represents the distribution density of the data. wAMD: Wet AMD group (CNV and PCV together).

Totally 17 significantly differential metabolites were identified between patients with wAMD and controls (Figures 2, 3 and Table 2). These metabolites included six oxidized lipids ((±)12-HEPE, (±)12-HETE, (±)4-HDHA, (±)9-HETE, 14(S)-HDHA and 15-oxoETE), two benzene and substituted derivatives (2,4-dihydroxybenzoic acid and 1,2,3-trihydroxybenzene), two organic acid and its derivatives (1-methyluric acid and carbamoyl phosphate), two amino acid metabolomics (trimethylamine N-oxide and L-tryptophanamide), two nucleotide metabolomics (1-methylxanthine and UDP-glucose), 1-aminopropan-2-ol, 2-methoxybenzoic acid and vitamin D3. Except for UDP-glucose and carbamoyl phosphate, which presented lower concentrations in wAMD, the other metabolites showed higher concentrations in wAMD, suggesting that these metabolites accumulate in plasma under wAMD conditions.

**Table 2:** Differential metabolites in wAMD and subtypes.

**Group**	**Index**	**Compounds**	**Class**	**VIP**	***P* value**	**Fold_change**	**Log2FC**	**Type**
wAMD vs control (total=17, down- regulated=2, up- regulated=15)	MEDP831	1-Aminopropan-2-ol	Alcohol	1.0641	9.34×10^-5^	2.3339	1.2227	up
	MEDP084	Trimethylamine N-Oxide	Amino acid metabolomics	1.2276	7.70×10^-5^	2.3760	1.2485	up
	MEDP891	L-Tryptophan amide	Amino acid metabolomics	1.2183	8.39×10^-4^	2.2378	1.1621	up
	MEDN481	2,4-Dihydroxybenzoic Acid	Benzene and substituted derivatives	1.1529	3.80×10^-5^	2.1539	1.1070	up
	MEDN576	1,2,3-Trihydroxybenzene	Benzene and substituted derivatives	1.1270	4.92×10^-2^	2.2642	1.1790	up
	MEDN090	2-Methoxybenzoic Acid	Benzoic acid and its derivatives	1.9407	2.01×10^-5^	2.4212	1.2757	up
	MEDN241	Vitamin D3	Coothers enzyme factor and vitamin	2.0421	2.59×10^-21^	2.3674	1.2433	up
	MEDN139	1-Methylxanthine	Nucleotide metabolomics	1.3305	1.80×10^-5^	3.9526	1.9828	up
	MEDN538	UDP-glucose	Nucleotide metabolomics	1.4315	7.78×10^-8^	0.3640	-1.4581	down
	MEDN472	1-Methyluric Acid	Organic acid and its derivatives	1.2168	1.24×10^-5^	2.8901	1.5311	up
	MEDN615	Carbamoyl phosphate	Organic acid and its derivatives	1.1495	4.84×10^-6^	0.4812	-1.0554	down
	MEDN750	(±)12-HEPE	Oxidized lipid	1.0385	2.53×10^-2^	3.3824	1.7581	up
	MEDN751	(±)12-HETE	Oxidized lipid	1.0996	4.56×10^-2^	9.0656	3.1804	up
	MEDN758	(±)4-HDHA	Oxidized lipid	1.4334	9.30×10^-3^	2.8155	1.4934	up
	MEDN763	(±)9-HETE	Oxidized lipid	1.5778	1.73×10^-2^	2.0244	1.0175	up
	MEDN769	14(S)-HDHA	Oxidized lipid	1.5465	4.22×10^-2^	4.3538	2.1223	up
	MEDN771	15-oxoETE	Oxidized lipid	2.1349	3.93×10^-8^	3.3391	1.7394	up
CNV vs control total=14, down- regulated=3, up- regulated=11)	MEDP831	1-Aminopropan-2-ol	Alcohol	1.0634	8.85×10^-4^	2.6875	1.4263	up
	MEDP084	Trimethylamine N-Oxide	Amino acid metabolomics	1.1474	9.40×10^-4^	2.6220	1.3906	up
	MEDP891	L-Tryptophan	Amino acid metabolomics	1.6438	1.76×10^-3^	3.1248	1.6437	up
	MEDN553	2-Methylbenzoic acid	Benzene and substituted derivatives	1.0011	4.16×10^-2^	4.9730	2.3141	up
	MEDP697	2,6-Di-tert-butyl-4-methylphenol	Benzene and substituted derivatives	1.4278	1.20×10^-4^	2.0703	1.0498	up
	MEDN090	2-Methoxybenzoic Acid	Benzoic acid and its derivatives	1.5343	2.22×10^-3^	2.7788	1.4745	up
	MEDN241	Vitamin D3	Co others enzyme factor and vitamin	1.7297	5.56×10^-26^	2.9489	1.5602	up
	MEDN352	O-Phosphorylethanolamine	Lipids’ others phospholipid	1.3483	1.96×10^-7^	0.4769	-1.0681	down
	MEDN538	UDP-glucose	Nucleotide metabolomics	1.3909	3.68×10^-8^	0.3373	-1.5679	down
	MEDP881	Phosphocholine	Nucleotide metabolomics	2.4872	1.92×10^-26^	2.1194	1.0837	up
	MEDN472	1-Methyluric Acid	Organic acid and its derivatives	1.0114	8.89×10^-4^	2.5362	1.3427	up
	MEDN615	Carbamoyl phosphate	Organic acid and its derivatives	1.0599	2.48×10^-6^	0.4564	-1.1315	down
	MEDN758	(±)4-HDHA	Oxidized lipid	1.2348	4.54×10^-2^	3.5047	1.8093	up
	MEDN771	15-oxoETE	Oxidized lipid	1.5185	7.42×10^-6^	3.2664	1.7077	up
PCV vs control (total=9, down- regulated=1, up- regulated=8)	MEDP084	Trimethylamine N-Oxide	Amino acid metabolomics	1.0250	2.13×10^-2^	2.1013	1.0713	up
	MEDP087	L-Alanyl-L-Lysine	Amino acid metabolomics	2.7540	4.26×10^-13^	2.2845	1.1919	up
	MEDN090	2-Methoxybenzoic Acid	Benzoic acid and its derivatives	1.8151	2.83×10^-7^	2.0218	1.0157	up
	MEDN139	1-Methylxanthine	Nucleotide metabolomics	1.4939	8.86×10^-4^	4.7586	2.2505	up
	MEDN538	UDP-glucose	Nucleotide metabolomics	2.4156	2.37×10^-7^	0.3938	-1.3446	down
	MEDN472	1-Methyluric Acid	Organic acid and its derivatives	1.2840	1.45×10^-3^	3.2852	1.7160	up
	MEDN758	(±)4-HDHA	Oxidized lipid	1.4450	2.06×10^-3^	2.0459	1.0327	up
	MEDN771	15-oxoETE	Oxidized lipid	2.3710	3.91×10_-6_	3.4202	1.7741	up
	MEDN647	Hydroquinone	Phenols acid and its derivatives	1.5979	7.32×10^-4^	2.0060	1.0043	up
CNV vs PCV(total=2, down-regulated=2)	MEDP891	L-Tryptophan amide	Amino acid metabolomics	2.1151	6.25×10^-3^	0.3992	-1.3248	down
	MEDN109	Hyodeoxycholic Acid	Bile Acids	1.3908	3.14×10^-3^	0.4805	-1.0575	down

An index is a number set for the detected metabolites; total represents the number of screened differential metabolites; up-regulated represents the number of differential metabolites with increased relative content; down-regulated represents the number of differential metabolites with decreased relative content. Note: Index refers to the index we set for each metabolite detected. total= total sigmetabolites; up-regulated=The number of up-regulated metabolites; down-regulated=The number of down-regulated metabolites.

### Metabolites with significant differences in CNV vs control

In total, 14 significantly differential metabolites were found in the CNV group compared with the control group; most of them are also contributed to wAMD (Figures 2, 3 and Table 2). These metabolites include (±)4-HDHA and 15-oxoETE (oxidized lipids), 2-Methylbenzoic acid and 2,6-Di-tert-butyl-4-methylphenol (benzene and substituted derivatives), 1-methyluric acid and carbamoyl phosphate (organic acid and its derivatives), trimethylamine N-oxide and L-tryptophanamide (amino acid metabolomics), UDP-glucose and phosphocholine (nucleotide metabolomics), 1-aminopropan-2-ol (alcohol), 2-methoxybenzoic acid (benzoic acid and its derivatives), vitamin D3 (Coothers enzyme factor and vitamin) and O-phosphorylethanolamine (lipids' others phospholipid). Except for O-phosphorylethanolamine, UDP-glucose and carbamoyl phosphate are down-regulated in CNV, and the others are up-regulated in CNV.

### Metabolites with significant differences in PCV vs control

In total, nine significantly differential metabolites were found in patients with PCV compared with the controls; most of them also contributed to wet AMD (Figures 2, 3 and Table 2). The following three metabolites were specifically detected in patients with PCV: 1-methylxanthine (nucleotide metabolomics), hydroquinone (phenols and their derivatives) and L-alanyl-L-lysine (amino acid metabolomics). The rest were shared with patients with CNV, suggesting their close relationship at the metabolic level.

### Metabolites with significant differences in CNV vs PCV

When comparing CNV to PCV, two significantly differential metabolites were identified: hyodeoxycholic acid (bile acids) and L-tryptophanamide (amino acid metabolomics) (Figures 2, 3 and Table 2). Both of them accumulate in PCV.

### Pathway analysis of differential metabolites

**Figure 4:**
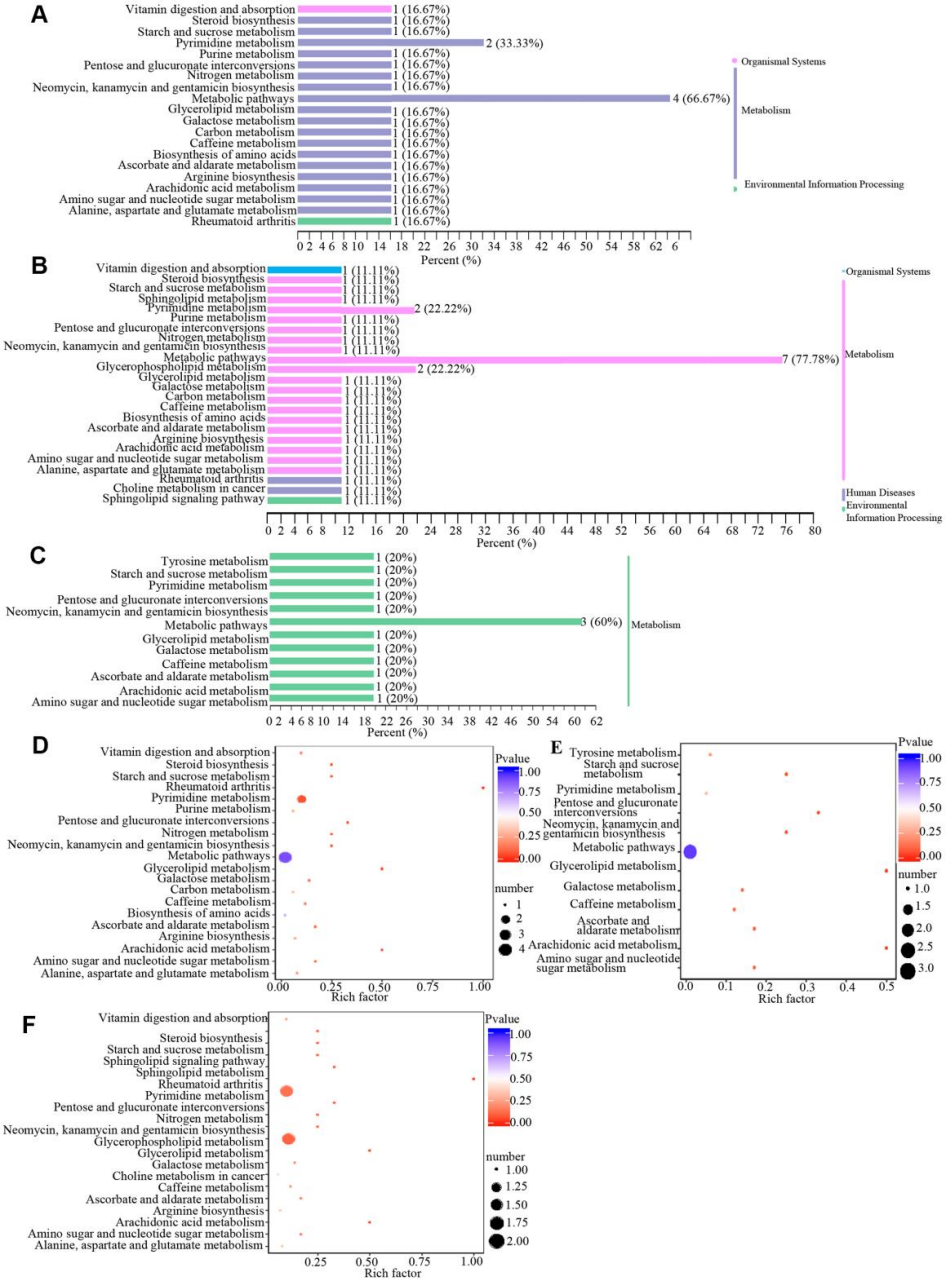
**Classification and enrichment of KEGG pathways of differential metabolites in wAMD, CNV, and PCV groups.** (**A**–**C**) are KEGG classification diagrams of differential metabolites of wAMD vs control, CNV vs control, and PCV vs control, respectively. The ordinate is the name of the KEGG metabolic pathway, and the abscissa is the number of metabolites from the annotation to the pathway and the proportion of the number of metabolites to the total number of annotated metabolites. (**D**–**F**) are the KEGG enrichment analysis graphs of differential metabolites wAMD vs control, PCV vs control, and CNV vs control. The rich factor is the ratio of the number of metabolites in the corresponding pathway to the total number of metabolites detected and annotated in the pathways. The larger the value is, the greater the enrichment degree is. The closer the *p*-value is to 0, the more significant the enrichment is. The size of the midpoint represents the number of significant metabolites enriched in the corresponding pathway.

Metabolites may interact with each other to form different pathways. By using KEGG annotation of the differential metabolites [39], metabolites were classified according to the type of pathway in KEGG (Figure 4 and Table 3). These results showed that metabolic pathways involved in metabolites included vitamin digestion and absorption, pyrimidine metabolism, biosynthesis, metabolic pathway, glycerophospholipid metabolism and other pathways.

**Table 3:** KEGG annotation results for differential metabolites.

**Group**	**Index**	**Compounds**	**cpd_ID**	**kEGG_map**
control vs wAMD	MEDN090	2-Methoxybenzoic Acid	--	--
	MEDN139	1-Methylxanthine	--	--
	MEDN241	Vitamin D3	C05443	ko00100,ko01100,ko04977,ko05323
	MEDN472	1-Methyluric Acid	C16359	ko00232
	MEDN481	2,4-Dihydroxybenzoic Acid	--	--
	MEDN538	UDP-glucose	C00029	ko00040,ko00052,ko00053,ko00240,ko00500,ko00520,ko00524,ko00561,ko01100
	MEDN576	1,2,3-Trihydroxybenzene	--	--
	MEDN615	Carbamoyl phosphate	C00169	ko00220,ko00230,ko00240,ko00250,ko00910,ko01100,ko01200,ko01230
	MEDN750	(±)12-HEPE	--	--
	MEDN751	(±)12-HETE	--	--
	MEDN758	(±)4-HDHA	--	--
	MEDN763	(±)9-HETE	--	--
	MEDN769	14(S)-HDHA	--	--
	MEDN771	15-oxoETE	C04577	ko00590
	MEDP084	Trimethylamine N-Oxide	C01104	ko01100
	MEDP831	1-Aminopropan-2-ol	--	--
	MEDP891	L-Tryptophan amide	--	--
control vs CNV	MEDN090	2-Methoxybenzoic Acid	--	--
	MEDN241	Vitamin D3	C05443	ko00100,ko01100,ko04977,ko05323
	MEDN352	O-Phosphorylethanolamine	C00346	ko00564,ko00600,ko01100,ko04071
	MEDN472	1-Methyluric Acid	C16359	ko00232
	MEDN538	UDP-glucose	C00029	ko00040,ko00052,ko00053,ko00240,ko00500,ko00520,ko00524,ko00561,ko01100
	MEDN553	2-Methylbenzoic acid	C07215	ko01100
	MEDN615	Carbamoyl phosphate	C00169	ko00220,ko00230,ko00240,ko00250,ko00910,ko01100,ko01200,ko01230
	MEDN758	(±)4-HDHA	--	--
	MEDN771	15-oxoETE	C04577	ko00590
	MEDP084	Trimethylamine N-Oxide	C01104	ko01100
	MEDP697	2,6-Di-tert-butyl-4-methylphenol	--	--
	MEDP831	1-Aminopropan-2-ol	--	--
	MEDP881	Phosphocholine	C00588	ko00564,ko01100,ko05231
	MEDP891	L-Tryptophanamide	--	--
control vs PCV	MEDN090	2-Methoxybenzoic Acid	--	--
	MEDN139	1-Methylxanthine	--	--
	MEDN472	1-Methyluric Acid	C16359	ko00232
	MEDN538	UDP-glucose	C00029	ko00040,ko00052,ko00053,ko00240,ko00500,ko00520,ko00524,ko00561,ko01100
	MEDN647	Hydroquinone	C00530	ko00350,ko01100
	MEDN758	(±)4-HDHA	--	--
	MEDN771	15-oxoETE	C04577	ko00590
	MEDP084	Trimethylamine N-Oxide	C01104	ko01100
	MEDP087	L-Alanyl-L-Lysine	--	--
CNV vs PCV	MEDN109	Hyodeoxycholic Acid	--	--
	MEDP891	L-Tryptophan amide	--	--

Cpd_ ID is the code of the corresponding metabolite in KEGG database_ Map is the number of pathways involved in the corresponding metabolites in KEGG database. Note: Index refers to the Index we set for each metabolite detected; Cpd_ID represents the corresponding ID of each metabolite in the KEGG database; KEGG_map refers to the number of pathways in which each metabolite participates in the KEGG database.

### Metabolites with significant differences linked to genotypes of AMD major associated genes *CFH* and *HTRA1*

AMD is a multifactorial disease, and genetic components play an important role in the pathogenesis of the disease [10]. Previous studies [11, 12, 14, 15] have shown that *HTRA1* and *CFH* are two major genes for AMD. To determine whether there are differences in plasma metabolites among different genotypes, we tested the genotypes of *CFH* r800292 and *HTRA1* rs10490924 (both are in the haplotype of the susceptible loci of *CFH* and *HTRA1*) in participants and then analyzed the metabolites and their differences among alleles. In total, 12 differential metabolites were identified in this analysis.

### Metabolites with significant differences between genotypes of CFH rs800282

**Figure 5:**
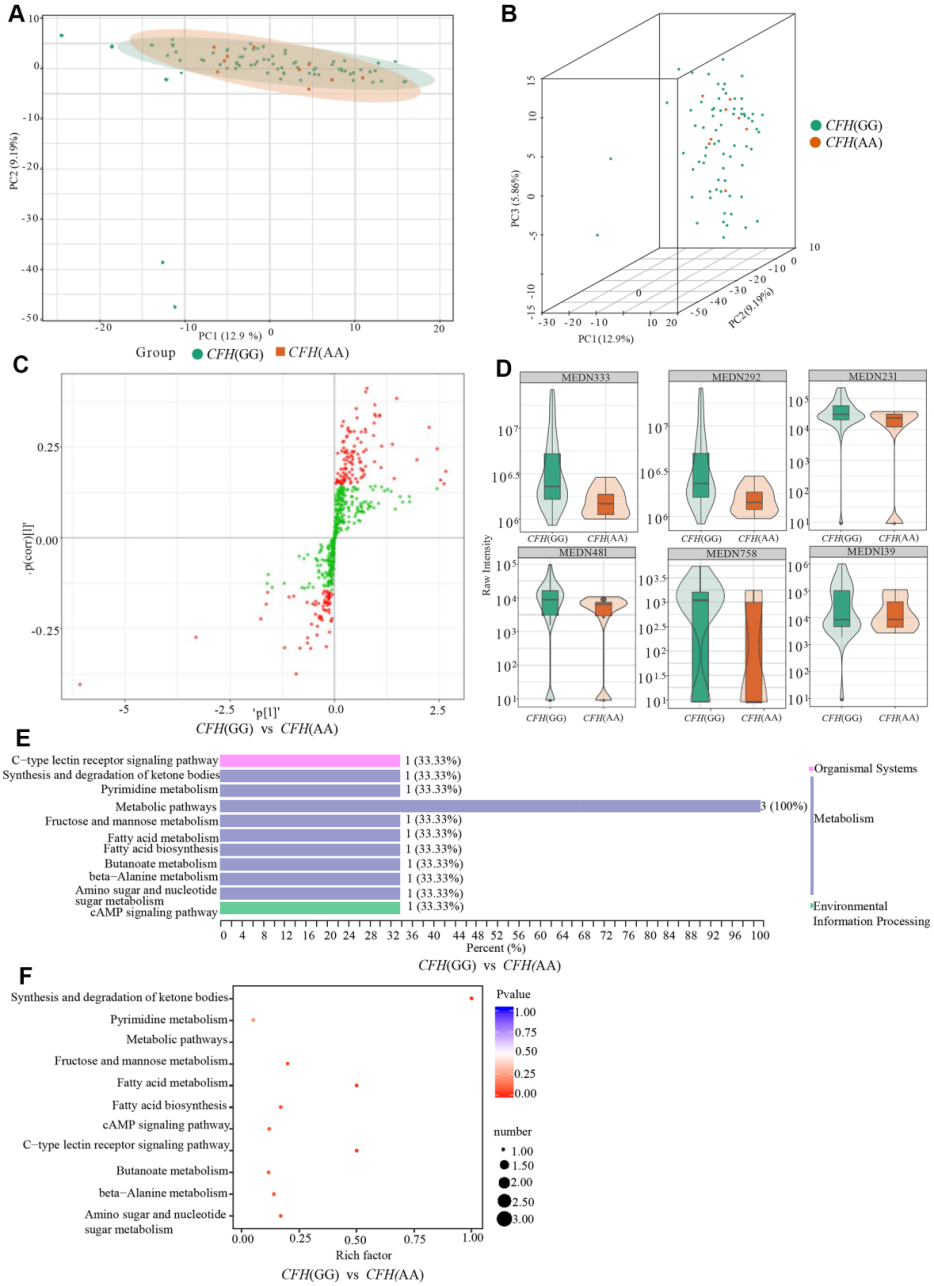
**Comparison of genotypes GG and AG of *CFH* rs800292.** (**A**) shows the two-dimensional PCA map of the degree of variation between the two groups of genotypes GG and AA, and (**B**) is the 3D images of PCA results of them. (**C**) is the OPLS-DA S-plot of *CFH* genotypes GG and AA. The abscissa represents the correlation coefficient of the principal component and metabolite, and the ordinate represents the correlation coefficient of the principal component and metabolite. The red dots indicate that the metabolites have VIP values greater than or equal to 1, and the green dots indicate that the metabolites have VIP values less than 1. (**D**) shows Relative contents of differential metabolites between *CFH* genotypes GG and AA. (**E**, **F**) are the results of KEGG classification and enrichment of differential metabolites between-group genotypes GG and group AA of *CFH*.

According to the PCA analysis results of metabolites detected in three genotypes of *CFH* rs800292 (Figure 5A, 5B), the degree of variation between genotypes is small, especially between genotype AA and genotype AG. The OPLS-DA S-plot was used to directly display the proportion of metabolites with VIP values greater than 1 or less than 1 in each group (Figure 5C). According to the screening criteria of differential metabolites, a total of six differential metabolites (1-methylxanthine, L-fucose, 3-hydroxybutyrate, malonic acid, 2,4-dihydroxybenzoic acid, (±)4-HDHA) were identified between genotypes GG and AA (Table 4 and Figure 5D). There were no significant metabolites between genotypes GG and AG. According to KEGG analysis, these six differential metabolites are mainly involved in the synthesis and degradation of ketone bodies, pyrimidine metabolism, fructose and mannose metabolism, fatty acid metabolism, fatty acid biosynthesis, the cAMP signaling pathway, and the C-type lectin receptor signaling pathway (Figure 5E, 5F and Table 4).

**Table 4:** Differential metabolites between GG and AA of *CFH* rs800292.

**Index**	**Compounds**	**Class**	**VIP**	***P* value**	**Fold_change**	**Log2FC**	**Type**		**cpd_ID**	**KEGG_map**
MEDN481	2,4-Dihydroxybenzoic Acid	Benzene and substituted derivatives	1.7005	7.01×10^-4^	0.4132	-1.2752	down		--	--
MEDN231	L-Fucose	Carbohydrate metabolomics	2.1101	4.25×10^-4^	0.4389	-1.1881	down		C01019	ko00051,ko00520,ko01100,ko04625
MEDN139	1-Methylxanthine	Nucleotide metabolomics	1.1322	1.09×10^-2^	0.3048	-1.7141	down		--	--
MEDN292	3-Hydroxybutyrate	Organic Acid And its Derivatives	2.3287	1.77×10^-5^	0.3617	-1.4673	down		C01089	ko00072,ko00650,ko01100,ko04024
MEDN333	Malonicacid	Organic Acid And its Derivatives	2.3457	1.92×10^-5^	0.3639	-1.4584	down		C00383	ko00061,ko00240,ko00410,ko01100,ko01212
MEDN758	(±)4-HDHA	Oxidized lipid	1.4931	2.06×10^-2^	0.4390	-1.1876	down		--	--

### Metabolites with significant differences between genotypes of HTRA1 rs10490924

**Figure 6:**
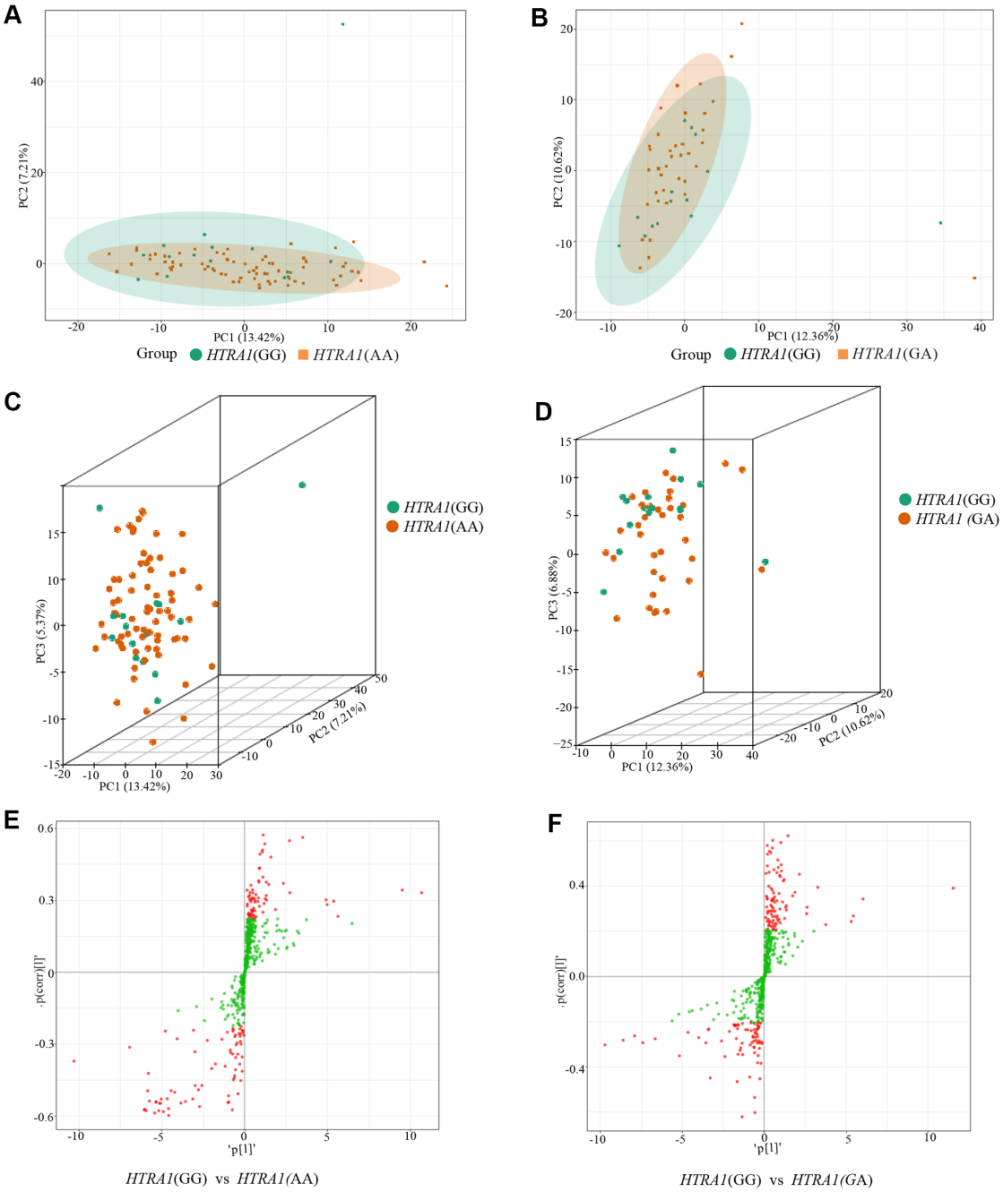
**PCA results and OPLS-DA S-plot of three *HTRA1* rs10490924 genotypes.** (**A**, **B**) show the two-dimensional PCA map of the degree of variation between the two groups of genotypes GG and AA, GG and GA. (**C**, **D**) are the three-dimensional PCA map. From the graphs, we can see that the degree of variation between genotypes GG and AA or between genotype GG and GA is relatively small. (**E**, **F**) are OPLS-DA S-plot of three *HTRA1* genotypes compared among groups. (**E**) shows the results of *HTRA1* genotypes GG and AA, and (**F**) shows the results of GG and GA. This diagram mainly shows the number of metabolites whose VIP value is greater than or less than 1 in the detected metabolites between groups. The abscissa represents the correlation coefficient of the principal component and metabolite, and the ordinate represents the correlation coefficient of the principal component and metabolite. The closer the metabolite is to the upper right corner and the lower-left corner, the more significant the difference is. The red dots indicate that the metabolites have VIP values greater than or equal to 1, and the green dots indicate that the metabolites have VIP values less than 1.

**Figure 7:**
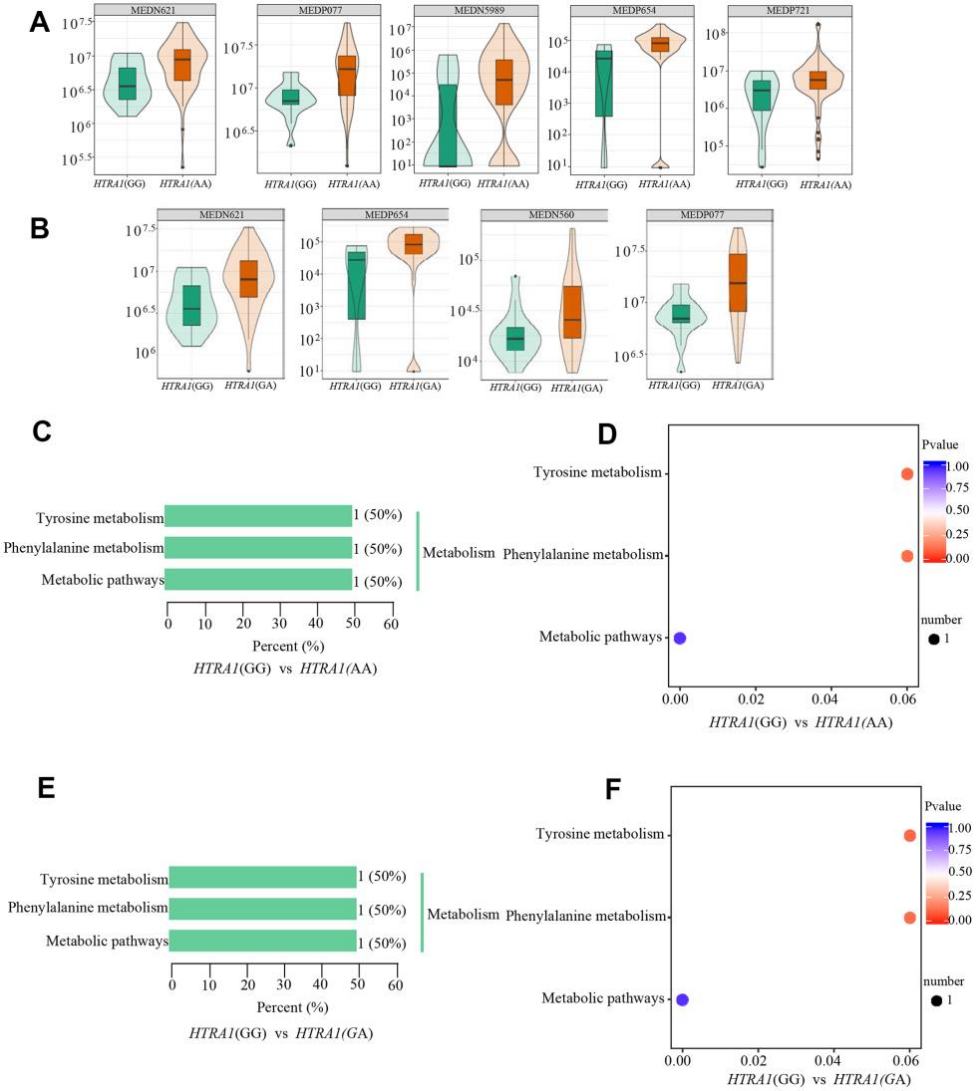
**Relative contents and the results of KEGG classification and enrichment of differential metabolites between *HTRA1* rs10490924 genotypes GG, AA, and GA.** (**A**, **B**) are relative contents of differential metabolites between *HTRA1* genotypes GG, AA, and GA. (**C**, **D**) are the results of KEGG classification and enrichment genotypes GG and AA. (**E**, **F**) are the results of KEGG classification and enrichment genotypes GG and GA. Because there are three kinds of repeated metabolites in the two groups, the classification and enrichment analysis results of KEGG are very similar.

Similar to *CFH*, the PCA analysis results suggested that three genotypes of *HTRA1* rs10490924 had small variations (Figure 6A–6D). The *HTRA1* OPLS-DA S-plot was used to visually display the proportion of metabolites with VIP values greater than 1 or less than 1 among groups (Figure 6E, 6F). According to the screening criteria of differential metabolites, five differential metabolites (cyclamic acid, indoxylsulfuric acid, phenylacetyl-L-glutamine, 3-indolepropionic acid, 2-phenylacetamide) were identified between GG and AA (Table 5 and Figure 7A, 7B). The relative contents of these five metabolites in *HTRA1* genotype AA were higher than those in group GG. Four differential metabolites (marmesin, indoxylsulfuric acid, phenylacetyl-L-glutamine, and 2-phenylacetamide) were identified between genotypes GG and AG. KEGG analysis showed that three of these metabolites were repetitive and were closely related to tyrosine metabolism, phenylalanine metabolism, and metabolic pathways (Table 5 and Figure 7C–7F).

**Table 5:** Differential metabolites between GG, AA and GA of the *HTRA1* rs10490924.

**Group**	**Index**	**Compounds**	**Class**	**VIP**	***P* value**	**Fold_change**	**Log2FC**	**Type**	**cpd_ID**	**KEGG_map**
GG vs AA	MEDP077	Phenylacetyl-L-Glutamine	Amino acid metabolomics	1.7093	6.13×10^-8^	2.3134	1.2100	up	C05595	ko00350
	MEDP654	2-Phenylacetamide	Benzene and substituted derivatives	1.0977	7.66×10^-8^	3.2073	1.6813	up	C02505	ko00360, ko01100
	MEDP271	3-Indolepropionic Acid	Indole and its derivatives	1.4822	1.09×10^-2^	3.6082	1.8513	up	-	-
	MEDN589	Cyclamic acid	Organic acid and its derivatives	1.6573	1.30×10^-2^	9.8740	3.3036	up	-	-
	MEDN621	Indoxylsulfuric acid	Organic acid and its derivatives	1.7961	1.01×10^-4^	2.0235	1.0168	up	-	-
GG vs GA	MEDP077	Phenylacetyl-L-Glutamine	Amino acid metabolomics	2.2041	3.75×10^-5^	2.3749	1.2478	up	C05595	ko00350
	MEDP654	2-Phenylacetamide	Benzene and substituted derivatives	1.3055	2.04×10^-5^	3.3779	1.7561	up	C02505	ko00360, ko01100
	MEDN560	Marmesin	Carbohydrate metabolomics	1.2517	1.23×10^-2^	2.0677	1.0480	up	-	-
	MEDN621	Indoxylsulfuric acid	Organic acid and its derivatives	1.8129	9.93×10^-4^	2.0431	1.0308	up	-	-

### Metabolites of microorganisms

To explore whether the metabolites of microorganisms participate in the occurrence of wAMD, we noted the 545 metabolites detected in patients in the METLIN database (https://metlin.scripps.edu) and identified 24 microbial-specific metabolites (Table 6 and Supplementary File 3), most of which are organic acids and their derivatives (*N* = 6, 25.0%), followed by benzene and its substituted derivatives (*N* = 4, 16.67%). However, these metabolites did not show significant differences between patients and controls. Among them, we found that the cyclamic acid concentration was different between genotypes GG and AA of *HTRA1* rs10490924 (*P* = 0.01, VIP = 1.66, fold change = 9.87), and its relative concentration of genotype AA was higher than that of genotype GG.

**Table 6:** Twenty-four microorganisms metabolites.

**Index**	**Compounds**	**Class**	**cpd_ID**
MEDP831	1-Aminopropan-2-ol	Alcohol	C05771
MEDP844	furfuryl alcohol	Alcohol	C20441
MEDP716	cis-Citral	Aldehyde	C09847
MEDP672	Cyclohexylamine	Amines	C00571
MEDN576	1,2,3-Trihydroxybenzene	Benzene and substituted derivatives	C01108
MEDP102	Syringic Acid	Benzene and substituted derivatives	C10833
MEDP111	3-(4-Hydroxyphenyl)-Propionic Acid	Benzene and substituted derivatives	C01744
MEDP796	Pyrene	Benzene and substituted derivatives	C14335
MEDN228	D-Arabinose	Carbohydrate metabolomics	-
MEDN625	Formononetin	Carbohydrate metabolomics	C00858
MEDN679	Maltol	Heterocyclic compound	C11918
MEDP546	Oxindole	Indole and its derivatives	C12312
MEDP799	(-)-Menthone	Ketones	C00843
MEDP839	Pulegone	Ketones	C09893
MEDP561	Farnesene	Lipids_fatty acids	C09665
MEDN334	Mandelic Acid	Organic acid and its derivatives	C01984
MEDN338	Phenyllactate (Pla)	Organic acid and its derivatives	C01479
MEDN346	Vanillic Acid	Organic acid and its derivatives	C06672
MEDN589	Cyclamic acid	Organic acid and its derivatives	C02824
MEDP303	Chlorogenic Acid	Organic acid and its derivatives	C00852
MEDN654	3-Methylsalicylic acid	Organic acid and its derivatives	C14088
MEDP130	4-Nitrophenol	Phenols and its derivatives	C00870
MEDP668	m-Cresol	Phenols and its derivatives	C01467
MEDP791	4-aminophenol	Phenols and its derivatives	C02372

### *In vitro* functional validation

Of the discovered differential metabolites, hyodeoxycholic acid and L-tryptophanamide are the only two differential metabolites between CNV and PCV. Cyclamic acid is one of the unique metabolites of microorganisms that can inhibit intercellular communication [40] and affect cell morphology [41]. The relative content of cyclamic acid in the AMD risk genotype AA was higher than that in the protective genotype GG in rs10490924 (HTRA1 locus) [15]. O-phosphoethanolamine is a protective metabolite for CNV group. O-phosphoethanolamine is involved in the metabolism of glycerophospholipids and sphingolipids, and is associated with Alzheimer's disease, a degenerative disease that shared some common genetic variants with AMD [42–45]. Therefore, to further explore the effects of these differential metabolites, we selected cyclamic acid, hyodeoxycholic acid, L-tryptophanamide, and O-phosphorylethanolamine on, for testing their effects on human retinal pigment epithelium cells (HRPECs) and primary human retinal endothelial cells (HRECs) which are highly related with the pathogenesis of AMD.

### Effects on HRPECs

**Figure 8:**
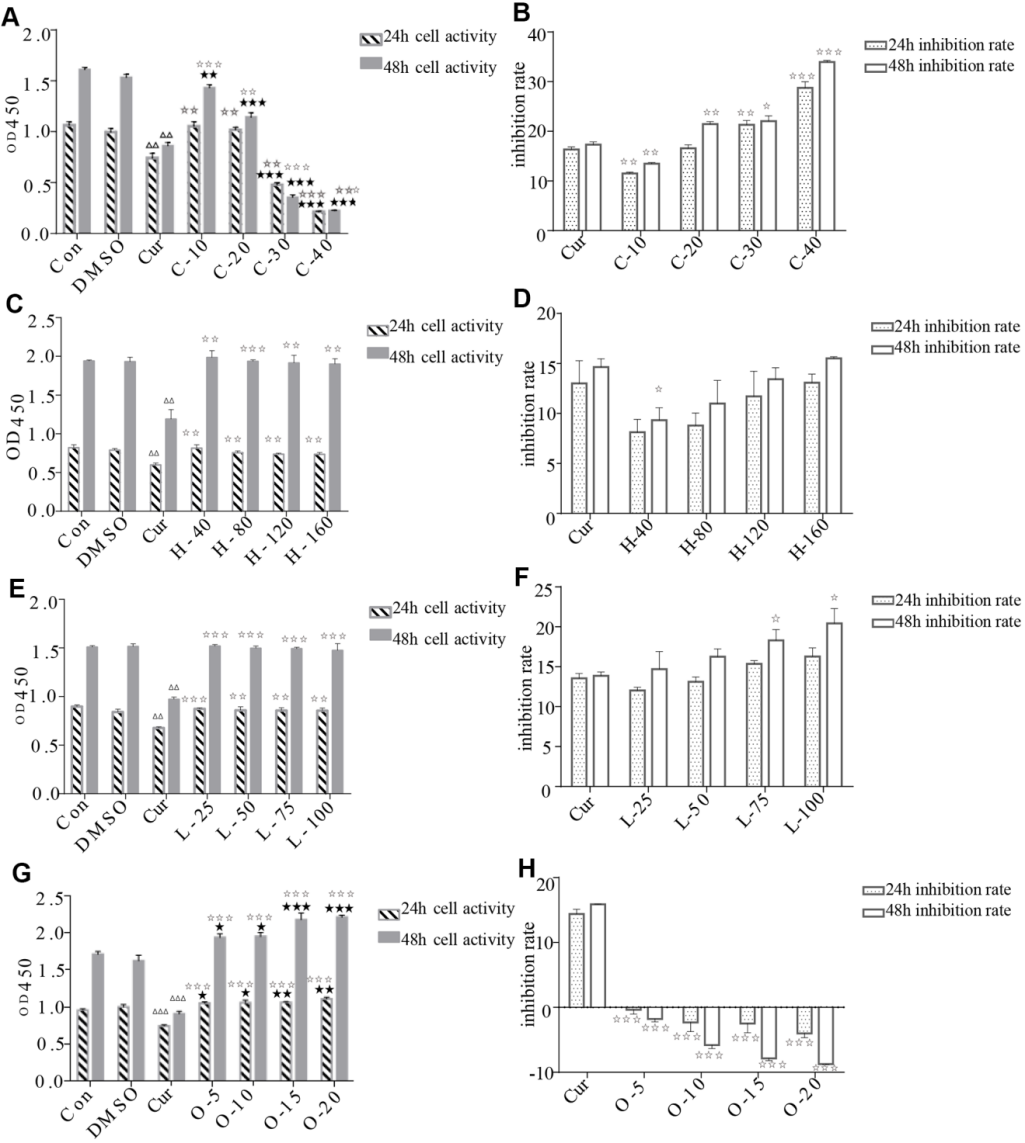
**Effects of cyclamic acid, hyodeoxycholic acid, L-tryptophanamide, and O-phosphorylethanolamine on the activity and proliferation of HRPECs.** (**A**, **C**, **E**, **G**) respectively reflected the changes of cell activity after hRPE cells were treated with cyclamic acid, hyodeoxycholic acid, L-tryptophanamide, and O-phosphorylethanolamine. (**B**, **D**, **F**, **H**) respectively reflected the changes of cell proliferation inhibition rate after treatment. C-10, C-20, C-30 and C-40 represent 10 μmol/ml, 20 μmol/ml, 30 μmol/ml and 40 μmol/ml cyclamic acid, respectively. H-40, H-80, H-120 and H-160 represent 40 μM, 80 μM, 120 μM, 160 μM hyodeoxycholic acid, respectively. L-25, L-50, L-75 and L-100 represent 25 μM, 50 μM, 75 μM and 100 μM L-tryptophanamide. O-5, O-10, O-15 and O-20 represent 5 μmol/ml, 10 μmol/ml, 15 μmol/ml and 20 μmol/ml O-phosphorylethanolamine. ΔΔ and ΔΔΔ indicated that the positive control group (cur) was significantly different from the 0.1% ✰ ✰ DMSO group (0.001<p<0.01, p<0.001). ✰ and ✰✰✰ respectively indicated that there were significant differences between this group and the positive control group, and the corresponding values were 0.01<p<0.05, 0.001<p<0.01, p<0.001.⋆, ⋆ ⋆ and ⋆⋆⋆ respectively indicated that there were significant differences between this group and the blank control group (con), and the corresponding values were 0.01<p<0.05, 0.001<p<0.01, p<0.001.

**Figure 9:**
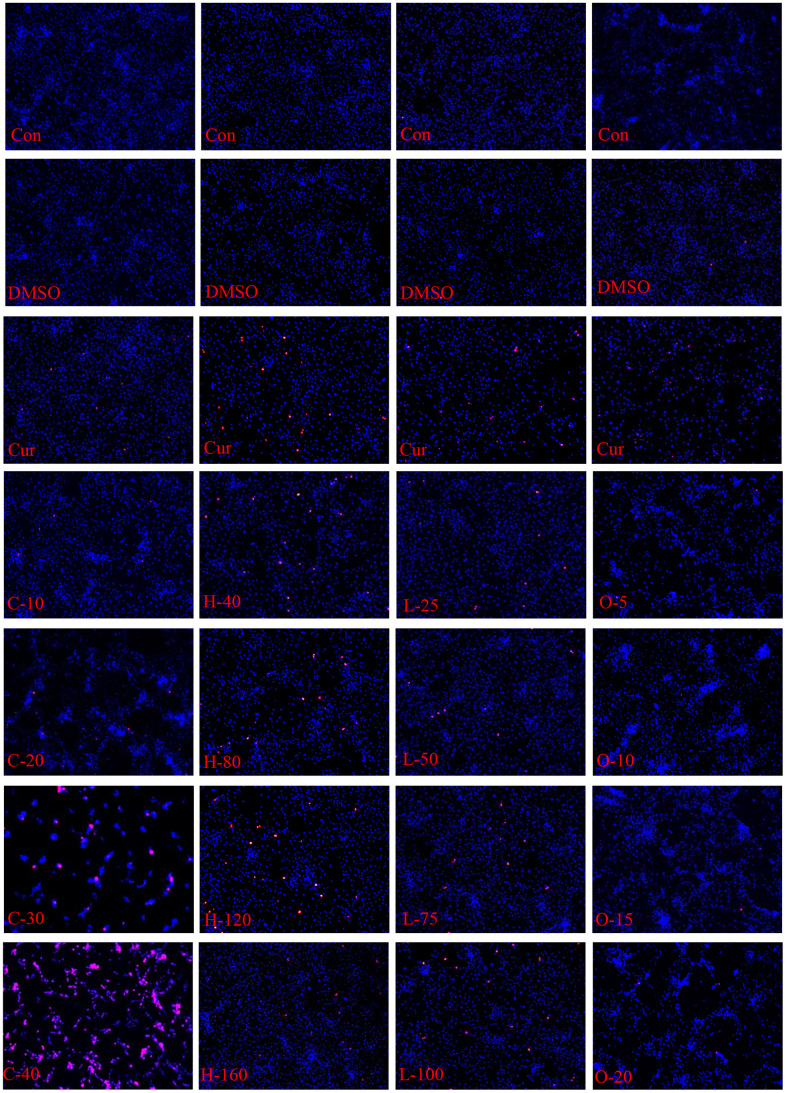
**Apoptosis and necrosis of HRPECs.** The apoptosis and necrosis of HRPECs after being treated with cyclamic acid, hyodeoxycholic acid, L-tryptophanamide, and O-phosphorylethanolamine for 48h. Blue fluorescence shows normal cells, bright blue shows apoptotic cells, and red shows necrotic cells.

The results of CCK-8 cell proliferation and cytotoxicity assay of the four metabolites and controls in HRPECs are presented in Figure 8 and Supplementary File 4. Treatment of HRPECs with 30μmol/ml and 40μmol/ml of cyclamic acid for 24h and 48h significantly inhibited the activity of HRPECs comparing to curcumin, a positive control (Figure 8A and Supplementary File 4). The proliferation of HRPECs was significantly inhibited by 10μmol/ml and 20μmol/ml cyclamic acid for 48h (Figure 8B and Supplementary File 4). Hyodeoxycholic acid and L-tryptophanamide had no significant effect on the activity of HRPECs comparing to curcumin (Figure 8C–8E and Supplementary File 4). However, after 48h treatment of L-tryptophanamide, the proliferation inhibition rate of HRPECs increased comparing to curcumin (Figure 8F and Supplementary File 4). O-phosphorylethanolamine increased the activity of HRPECs comparing to curcumin suggesting a promote function for the proliferation of HRPECs (Figure 8G, 8H and Supplementary File 4). The results of apoptosis and necrosis assay after treating the four selected metabolites in HRPECs were presented in Figure 9 and Tables 7–10. Cyclamic acid treatment of HRPECs increased cells’ apoptosis and necrosis comparing with curcumin (Figure 9 and Table 7). The effects of hyodeoxycholic acid, L-tryptophanamide and O-phosphorylethanolamine on HRPECs are in between curcumin and DMSO (Figure 9 and Tables 8–10).

**Table 7:** The effects of cyclamic acid on apoptosis and necrosis of HRPECs.

	**Group**	**Mean±SEM (%)**	**Comparing group**	***P* value**
Apoptosis rate (24h)	Con	4.745 ± 0.167	Con vs DMSO	0.956
	DMSO	4.774 ± 0.451	DMSO vs Cur	0.002
	Cur	11.960 ± 0.844	C-10 vs Con	0.107
	C-10	5.273 ± 0.193	C-20 vs Con	0.012
	C-20	6.011 ± 0.238	C-30 vs Con	0.005
	C-30	6.274 ± 0.208	C-40 vs Con	0.000
	C-40	1.524 ± 0.154	C-10 vs Cur	0.002
			C-20 vs Cur	0.003
			C-30 vs Cur	0.003
			C-40 vs Cur	0.000
Apoptosis rate (48h)	Con	6.937 ± 0.245	Con vs DMSO	0.571
	DMSO	7.518 ± 0.911	DMSO vs Cur	0.032
	Cur	12.190 ± 1.125	C-10 vs Con	0.923
	C-10	6.820 ± 1.105	C-20 vs Con	0.016
	C-20	8.952 ± 0.436	C-30 vs Con	0.000
	C-30	38.030 ± 1.714	C-40 vs Con	0.000
	C-40	0.196 ± 0.108	C-10 vs Cur	0.027
			C-20 vs Cur	0.055
			C-30 vs Cur	0.000
			C-40 vs Cur	0.000
Necrosis rate (24h)	Con	0.045 ± 0.045	Con vs DMSO	0.892
	DMSO	0.037 ± 0.037	DMSO vs Cur	0.000
	Cur	1.382 ± 0.051	C-10 vs Con	0.012
	C-10	0.590 ± 0.115	C-20 vs Con	0.002
	C-20	0.781 ± 0.085	C-30 vs Con	0.010
	C-30	5.889 ± 1.272	C-40 vs Con	0.025
	C-40	38.820 ± 11.150	C-10 vs Cur	0.003
			C-20 vs Cur	0.004
			C-30 vs Cur	0.024
Necrosis rate (48h)	Con	0.046 ± 0.023	Con vs DMSO	0.864
	DMSO	0.041 ± 0.020	DMSO vs Cur	0.048
	Cur	3.548 ± 1.248	C-10 vs Con	0.012
	C-10	0.615 ± 0.128	C-20 vs Con	0.006
	C-20	0.828 ± 0.141	C-30 vs Con	0.003
	C-30	9.430 ± 1.451	C-40 vs Con	0.000
	C-40	96.600 ± 1.589	C-10 vs Cur	0.080
			C-20 vs Cur	0.139
			C-30 vs Cur	0.040
			C-40 vs Cur	0.000

Con stands for control group, DMSO for 0.1% DMSO group, cur for curcumin group. C-10, C-20, C-30 and C-40 represent 10 μmol/ml, 20 μmol/ml, 30 μmol/ml and 40 μmol/ml cyclamic acid, respectively.

**Table 8:** The effects of hyodeoxycholic acid on apoptosis and necrosis of HRPECs.

	**Group**	**Mean±SEM (%)**	**Comparing group**	***P* value**
Apoptosis rate (24h)	Con	4.945 ± 0.147	Con vs DMSO	0.149
	DMSO	5.568 ± 0.317	DMSO vs Cur	0.001
	Cur	11.480 ± 0.677	H-40 vs DMSO	0.221
	H-40	4.974 ± 0.260	H-80 vs DMSO	0.607
	H-80	5.072 ± 0.832	H-120 vs DMSO	0.479
	H-120	5.133 ± 0.458	H-160 vs DMSO	0.055
	H-160	5.898 ± 2.684	H-40 vs Cur	0.001
			H-80 vs Cur	0.004
			H-120 vs Cur	0.002
			H-160 vs Cur	0.023
Apoptosis rate (48h)	Con	6.631 ± 0.072	Con vs DMSO	0.616
	DMSO	6.637 ± 0.565	DMSO vs Cur	0.001
	Cur	13.410 ± 1.915	H-40 vs DMSO	0.323
	H-40	5.047 ± 0.716	H-80 vs DMSO	0.054
	H-80	5.105 ± 0.558	H-120 vs DMSO	0.042
	H-120	5.222 ± 0.871	H-160 vs DMSO	0.005
	H-160	7.301 ± 0.533	H-40 vs Cur	0.002
			H-80 vs Cur	0.003
			H-120 vs Cur	0.004
			H-160 vs Cur	0.002
Necrosis rate (24h)	Con	0.057 ± 0.032	Con vs DMSO	0.992
	DMSO	0.095 ± 0.063	DMSO vs Cur	0.027
	Cur	1.300 ± 0.113	H-40 vs DMSO	0.156
	H-40	0.220 ± 0.091	H-80 vs DMSO	0.126
	H-80	0.351 ± 0.071	H-120 vs DMSO	0.244
	H-120	0.387 ± 0.077	H-160 vs DMSO	0.442
	H-160	0.565 ± 0.053	H-40 vs Cur	0.015
			H-80 vs Cur	0.014
			H-120 vs Cur	0.018
			H-160 vs Cur	0.037
Necrosis rate (48h)	Con	0.085 ± 0.014	Con vs DMSO	0.643
	DMSO	0.100 ± 0.027	DMSO vs Cur	0.001
	Cur	2.474 ± 0.252	H-40 vs DMSO	0.000
	H-40	1.257 ± 0.061	H-80 vs DMSO	0.000
	H-80	1.318 ± 0.059	H-120 vs DMSO	0.003
	H-120	1.458 ± 0.207	H-160 vs DMSO	0.002
	H-160	1.499 ± 0.199	H-40 vs Cur	0.009
			H-80 vs Cur	0.011
			H-120 vs Cur	0.036
			H-160 vs Cur	0.039

Con stands for the control group, DMSO for 0.1% DMSO group, cur for curcumin group. H-40, H-80, H-120 and H-160 represent 40 μM, 80 μM, 120 μM, 160 μM hyodeoxycholic acid, respectively.

**Table 9:** The effects of L-tryptophanamide on apoptosis and necrosis of HRPECs.

	**Group**	**Mean±SEM (%)**	**Comparing group**	***P* value**
Apoptosis rate (24h)	Con	4.733 ± 0.029	Con vs DMSO	0.601
	DMSO	4.395 ± 0.596	DMSO vs Cur	0.037
	Cur	8.232 ± 0.607	L-25 vs DMSO	0.096
	L-25	6.248 ± 0.611	L-50 vs DMSO	0.074
	L-50	6.945 ± 0.876	L-75 vs DMSO	0.049
	L-75	7.538 ± 0.948	L-100 vs DMSO	0.016
	L-100	8.034 ± 0.691	L-25 vs Cur	0.083
			L-50 vs Cur	0.294
			L-75 vs Cur	0.570
			L-100 vs Cur	0.840
Apoptosis rate (48h)	Con	7.635 ± 0.726	Con vs DMSO	0.843
	DMSO	7.845 ± 0.324	DMSO vs Cur	0.000
	Cur	10.650 ± 0.334	L-25 vs DMSO	0.079
	L-25	7.542 ± 0.161	L-50 vs DMSO	0.008
	L-50	7.944 ± 0.292	L-75 vs DMSO	0.007
	L-75	11.420 ± 0.777	L-100 vs DMSO	0.000
	L-100	13.050 ± 0.656	L-25 vs Cur	0.007
			L-50 vs Cur	0.110
			L-75 vs Cur	0.173
			L-100 vs Cur	0.052
Necrosis rate (24h)	Con	0.085 ± 0.044	Con vs DMSO	0.805
	DMSO	0.100 ± 0.055	DMSO vs Cur	0.004
	Cur	0.943 ± 0.046	L-25 vs DMSO	0.449
	L-25	0.372 ± 0.102	L-50 vs DMSO	0.832
	L-50	0.703 ± 0.108	L-75 vs DMSO	0.013
	L-75	0.744 ± 0.111	L-100 vs DMSO	0.002
	L-100	0.800 ± 0.025	L-25 vs Cur	0.001
			L-50 vs Cur	0.004
			L-75 vs Cur	0.415
			L-100 vs Cur	0.031
Necrosis rate (48h)	Con	0.110 ± 0.030	Con vs DMSO	0.801
	DMSO	0.104 ± 0.013	DMSO vs Cur	0.001
	Cur	3.216 ± 0.306	L-25 vs DMSO	0.008
	L-25	0.485 ± 0.076	L-50 vs DMSO	0.002
	L-50	0.820 ± 0.095	L-75 vs DMSO	0.000
	L-75	1.304 ± 0.075	L-100 vs DMSO	0.000
	L-100	1.970 ± 0.162	L-25 vs Cur	0.001
			L-50 vs Cur	0.002
			L-75 vs Cur	0.004
			L-100 vs Cur	0.023

Con stands for the control group, DMSO for 0.1% DMSO group, cur for the curcumin group. L-25, L-50, L-75 and L-100 represent 25 μM, 50 μM, 75 μM and 100 μM L-tryptophanamide.

**Table 10:** The effects of O-phosphorylethanolamine on apoptosis and necrosis of HRPECs.

	**Group**	**Mean±SEM (%)**	**Comparing group**	***P* value**
Apoptosis rate (24h)	Con	3.376 ± 0.133	Con vs DMSO	0.718
	DMSO	3.490 ± 0.262	DMSO vs Cur	0.000
	Cur	11.330 ± 0.173	O-5 vs Con	0.007
	O-5	4.682 ± 0.224	O-10 vs Con	0.005
	O-10	5.301 ± 0.324	O-15 vs Con	0.019
	O-15	6.817 ± 0.895	O-20 vs Con	0.000
	O-20	6.967 ± 0.245	O-5 vs Cur	0.000
			O-10 vs Cur	0.000
			O-15 vs Cur	0.008
			O-20 vs Cur	0.000
Apoptosis rate (48h)	Con	3.536 ± 0.139	Con vs DMSO	0.067
	DMSO	3.985 ± 0.113	DMSO vs Cur	0.000
	Cur	12.070 ± 0.059	5 vs Con	0.014
	O-5	4.701 ± 0.243	10 vs Con	0.028
	O-10	5.671 ± 0.620	15 vs Con	0.000
	O-15	6.903 ± 0.173	20 vs Con	0.013
	O-20	7.650 ± 0.947	O-5 vs Cur	0.000
			O-10 vs Cur	0.001
			O-15 vs Cur	0.000
			O-20 vs Cur	0.010
Necrosis rate (24h)	Con	0.101 ± 0.009	Con vs DMSO	0.893
	DMSO	0.094 ± 0.048	DMSO vs Cur	0.000
	Cur	1.133 ± 0.012	5 vs Con	0.219
	O-5	0.163 ± 0.042	10 vs Con	0.485
	O-10	0.165 ± 0.083	15 vs Con	0.171
	O-15	0.191 ± 0.054	20 vs Con	0.064
	O-20	0.228 ± 0.049	O-5 vs Cur	0.000
			O-10 vs Cur	0.000
			O-15 vs Cur	0.000
			O-20 vs Cur	0.000
Necrosis rate (48h)	Con	0.104 ± 0.027	Con vs DMSO	0.729
	DMSO	0.116 ± 0.018	DMSO vs Cur	0.000
	Cur	3.029 ± 0.052	50 vs Con	0.098
	O-5	0.166 ± 0.010	10 vs Con	0.307
	O-10	0.180 ± 0.059	15 vs Con	0.060
	O-15	0.210 ± 0.030	20 vs Con	0.132
	O-20	0.232 ± 0.062	O-5 vs Cur	0.000
			O-10 vs Cur	0.000
			O-15 vs Cur	0.000
			O-20 vs Cur	0.000

Con stands for control group, DMSO for 0.1% DMSO group, cur for curcumin group. O-5, O-10, O-15 and O-20 represent 5 μmol/ml, 10 μmol/ml, 15 μmol/ml and 20 μmol/ml O-phosphorylethanolamine.

### Effects on HRECs

**Figure 10:**
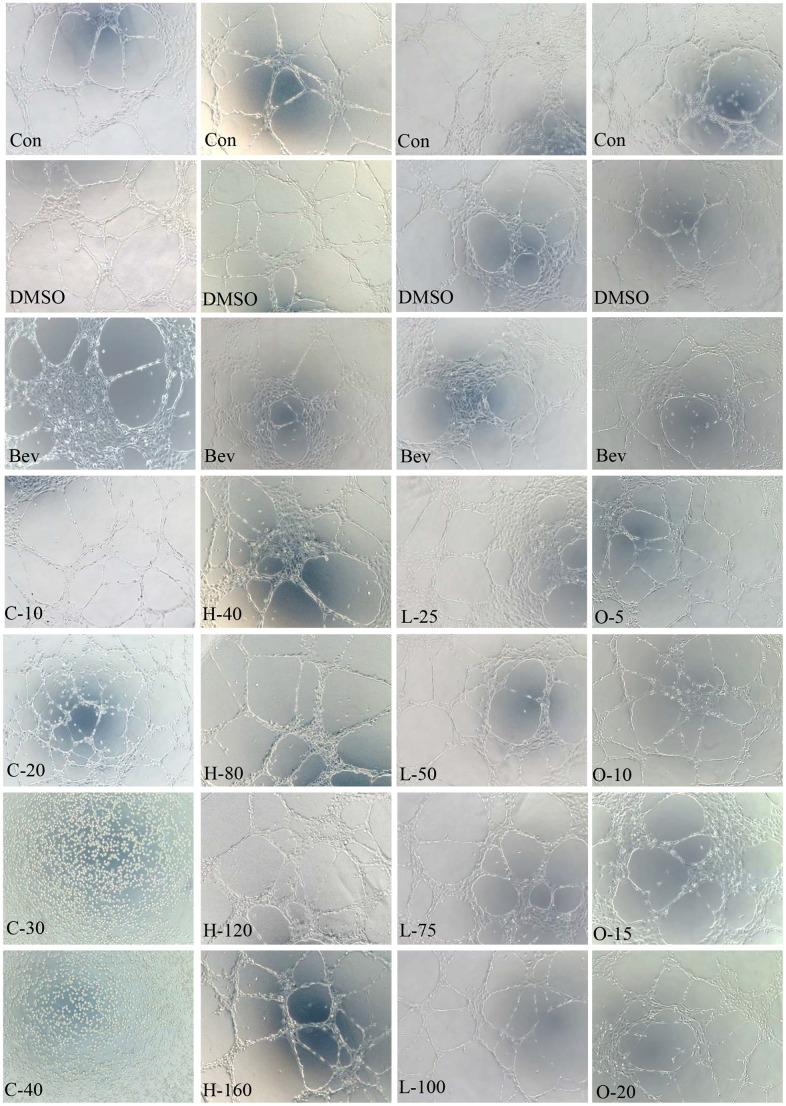
**HRECs tube formation.** Effects of cyclamic acid, hyodeoxycholic acid, L-tryptophanamide, and O-phosphorylethanolamine on the tubule formation of HRECs after treatment of 6h.

Angiogenesis is related to wAMD pathogenesis. Tube formation assay has been typically employed to demonstrate the angiogenic activity of vascular endothelial cells *in vitro*. We, therefore, performed the tube formation experiments by using HRECs (Figure 10 and Table 11). Cyclamic acid treated HRECs with 20 μmol/ml, the number of tubules increased comparing with the control group. However, tubules could not form after treated with higher concentrations (30μmol/ml and 40μmol/ml) (Figure 10 and Table 11), suggesting a concentration dependence effect. The number of tubules in HRECs treated with hyodeoxycholic acid had no difference with that in DMSO group, but was significantly higher than that in bevacizumab group, an inhibitor of vascular production (Figure 10 and Table 11). The number of tubules in HRECs treated with L-tryptophanamide was significantly more than that in DMSO and bevacizumab groups, suggesting a tube formation promote effect (Figure 10 and Table 11). HRECs treated with O-phosphorylethanolamine showed lower tube formation ability than that in control and bevacizumab groups, suggesting a tube formation inhibit effect (Figure 10 and Table 11).

**Table 11:** Statistics of branching points in tubule formation assay of HRECs.

**Group**	**Mean±SEM**	**Comparing group**	***P* value**
Con	48.000 ± 1.155	Con vs DMSO	0.067
DMSO	44.670 ± 0.667	Con vs Bev	0.001
Bev	37.000 ± 0.577	C-10 vs Con	0.340
C-10	45.000 ± 2.517	C-20 vs Con	0.000
C-20	75.330 ± 1.764	C-30 vs Con	0.000
C-30	0.000 ± 0.000	C-40 vs Con	0.000
C-40	0.000 ± 0.000	C-10 vs Bev	0.036
		C-20 vs Bev	0.000
		C-30 vs Bev	0.000
		C-40 vs Bev	0.000
Con	46.670 ± 0.333	Con vs DMSO	0.609
DMSO	46.000 ± 1.155	Con vs Bev	0.000
Bev	38.000 ± 0.577	H-40 vs DMSO	0.279
H-40	45.000 ± 0.577	H-80 vs DMSO	0.399
H-80	46.330 ± 0.333	H-120 vs DMSO	0.530
H-120	52.000 ± 1.732	H-160 vs DMSO	0.004
H-160	63.330 ± 1.202	H-40 vs Bev	0.001
		H-80 vs Bev	0.001
		H-120 vs Bev	0.002
		H-160 vs Bev	0.000
Con	46.670 ± 0.882	Con vs DMSO	0.368
DMSO	44.670 ± 1.764	Con vs Bev	0.001
Bev	37.000 ± 0.577	L-25 vs DMSO	0.250
L-25	41.000 ± 2.082	L-50 vs DMSO	0.899
L-50	45.000 ± 1.732	L-75 vs DMSO	0.057
L-75	50.330 ± 1.202	L-100 vs DMSO	0.001
L-100	62.670 ± 1.453	L-25 vs Bev	0.138
		L-50 vs Bev	0.012
		L-75 vs Bev	0.001
		L-100 vs Bev	0.000
Con	45.670 ± 0.333	Con vs DMSO	0.692
DMSO	45.000 ± 1.528	Con vs Bev	0.000
Bev	38.670 ± 0.333	O-5 vs Con	0.374
O-5	45.000 ± 0.577	O-10 vs Con	0.000
O-10	38.330 ± 0.333	O-15 vs Con	0.000
O-15	34.670 ± 0.882	O-20 vs Con	0.001
O-20	34.000 ± 1.155	O-5 vs Bev	0.001
		O-10 vs Bev	0.519
		O-15 vs Bev	0.013
		O-20 vs Bev	0.018

Wound healing assay has been used as an important tool to study cell polarization, tissue matrix rearrangement, and to predict cell proliferation and migration in HRECs. The wound-healing assay results of HRECs treated with the four selected metabolites are presented in Table 12. The migration rates of HRECs were significantly decreased after treatment with cyclamic acid comparing with bevacizumab. After hyodeoxycholic acid treatment, the migration rates were decreased compared with bevacizumab. After L-tryptophanamide treatment, the mobility increased comparing with DMSO. After 5 μmol/ml O-phosphorylethanolamine treatment with HRECs, the mobility was higher than that of bevacizumab, while after 10-20 μmol/ml O-phosphorylethanolamine treatment, the mobility was decreased compared with bevacizumab, suggesting a concentration dependence effect (Table 12).

**Table 12:** Migration rate of HRECs.

**Metabolites**	**Group**	**Mean±SEM**	**Comparing group**	***P* value**
Cyclamic acid	Con	60.256 ± 0.675	Con vs DMSO	0.849
	DMSO	60.441 ± 0.616	Con vs Bev	0.000
	Bev	47.897 ± 0.281	C-10 vs Con	0.000
	C-10	35.971 ± 1.161	C-20 vs Con	0.000
	C-20	6.367 ± 0.464	C-30 vs Con	0.000
	C-30	3.297 ± 0.170	C-40 vs Con	0.000
	C-40	2.589 ± 0.057	C-10 vs Bev	0.001
			C-20 vs Bev	0.000
			C-30 vs Bev	0.000
			C-40 vs Bev	0.000
Hyodeoxycholic acid	Con	59.621 ± 0.429	Con vs DMSO	0.670
	DMSO	59.070 ± 1.259	Con vs Bev	0.000
	Bev	47.936 ± 0.325	H-40 vs DMSO	0.787
	H-40	59.817 ± 2.261	H-80 vs DMSO	0.052
	H-80	55.416 ± 0.438	H-120 vs DMSO	0.001
	H-120	46.414 ± 0.066	H-160 vs DMSO	0.000
	H-160	38.208 ± 1.341	H-40 vs Bev	0.007
			H-80 vs Bev	0.000
			H-120 vs Bev	0.010
			H-160 vs Bev	0.002
L-Tryptophanamide	Con	59.688 ± 0.616	Con vs DMSO	0.974
	DMSO	59.718 ± 0.619	Con vs Bev	0.003
	Bev	47.100 ± 1.747	L-25 vs DMSO	0.106
	L-25	61.042 ± 0.139	L-50 vs DMSO	0.119
	L-50	61.933 ± 0.934	L-75 vs DMSO	0.042
	L-75	62.246 ± 0.596	L-100 vs DMSO	0.021
	L-100	63.925 ± 0.953	L-25 vs Bev	0.001
			L-50 vs Bev	0.002
			L-75 vs Bev	0.001
			L-100 vs Bev	0.001
O-Phosphorylethanolamine	Con	59.083 ± 0.741	Con vs DMSO	0.372
	DMSO	59.860 ± 0.227	Con vs Bev	0.000
	Bev	47.430 ± 0.412	O-5 vs Con	0.001
	O-5	50.880 ± 0.260	O-10 vs Con	0.000
	O-10	40.802 ± 0.715	O-15 vs Con	0.000
	O-15	15.243 ± 0.487	O-20 vs Con	0.000
	O-20	3.998 ± 0.067	O-5 vs Bev	0.002
			O-10 vs Bev	0.001
			O-15 vs Bev	0.000
			O-20 vs Bev	0.000

## DISCUSSION

By using UPLC and MS/MS, we investigated the different plasma metabolites between wAMD and normal people and between genotypes of AMD major associated genes *CFH* and *HTRA1*. These differential metabolites will provide potential targets for diagnosis and pathogenesis research of wAMD. The advantages of liquid-phase mass spectrometers are high sensitivity, wide dynamic range and no need for derivatization. LC-MS high-resolution metabolic profiling can be used to comprehensively evaluate up to 7000 plasma metabolites [46]. Standards and secondary spectra were used for the identification of metabonomics. Among them, the standard is the platinum standard for substance identification, and the analysis of secondary spectrum is the necessary data and technology for accurate identification of substances, so the quality is more accurate.

Most of the differentially up-regulated metabolites in plasma for wAMD vs normal controls were oxidized lipids, including (±)4-HDHA, (±)12-HEPE, (±)12-HETE, 14(S)-HDHA, (±)9-HETE, and 15-oxoETE. Among them, (±)12-HEPE, (±)12-HETE, (±)9-HETE and 15-oxoETE, are involved in arachidonic acid metabolism. Lipid oxide is the product of the oxidative stress reaction. Lipid oxide can produce oxidative stress itself and can also cause inflammatory reaction [47]. Oxidative stress plays an important role in the occurrence and development of wAMD [48], and antioxidants have a certain role in delaying the progress of CNV [49]. HDHA is a metabolite of omega-3 polyunsaturated fatty acids. It plays a role in the process of peroxisome proliferator-activated receptor γ (PPARγ), directly blocking endothelial cell proliferation and germinating angiogenesis, and is an effective direct inhibitor of vascular endothelial growth factor (VEGF)-induced CNV [50].

Vitamin D is the regulator of the immune system, which cooperates with *CFH* and *CFI* in the complement system and is involved in wAMD pathogenesis [51]. We found that there was a significant difference in vitamin D3 between the wAMD or CNV and controls. Previous studies [51, 52] showed that a vitamin D-rich diet can prevent or delay the occurrence and development of AMD, especially CNV. Vitamin D has also been shown to be antiangiogenic [53], which is involved in cell proliferation, differentiation and apoptosis [54]. In addition, vitamin D3 is also involved in steroid biosynthesis, vitamin digestion and absorption, and arthritis. The detailed role of vitamin D3 in wAMD pathogenesis is still to be further revealed.

Hyodeoxycholic acid and L-tryptophanamide were the only two differential metabolites in plasma of CNV group and PCV group, and their relative content in CNV group was higher than that in PCV group. In the experiment, it was found that hyodeoxycholic acid had no significant effect on HRPECs, but the migration rate of HRECs was significantly affected. L-tryptophanamide inhibited the proliferation of HRPECs, increased the necrosis rate of HRPECs, and promoted the formation and migration of HRPECs tubules. Therefore, L-tryptophanamide might damage HRPECs, promote the formation and migration of HRECs tubules, increase angiogenesis of CNV phenotype.

O-phosphorylethanolamine is involved in the metabolism of glycerophospholipids and sphingolipids. A study [55] found that other metabolites related to glycerophospholipids metabolism were low in AMD patients, such as diacylglycerol and phosphatidylcholine. In our experiment, we also found that the relative content of O-phosphoethanolamine in CNV patients was significantly lower than that in the control group. O-phosphoethanolamine significantly increased the activity of HRPECs, seemed to promote the proliferation of HRPECs, and inhibited the formation and migration of HRPECs tubules. O-phosphoethanolamine may play a protective role in the development of CNV, but more experiments are needed to explore whether it plays a role in preventing the occurrence of wet AMD.

A higher concentration of cyclamic acid was detected in the AA risk genotype than in the GG protective genotype of *HTRA1* rs10490924. Unabsorbed cyclamic acid can be metabolized into cyclohexylamine by intestinal microorganisms [56–59], and cyclohexylamine has greater toxicity [60]. According to the KEGG, both cyclamic acid and cyclohexylamine are involved in microbial metabolism in diverse environments (KEGG note: map01120). The common food additive sweetener- sodium cyclamate is similar to cyclamic acid. Morimoto's study found that sodium cyclamate can inhibit intercellular communication [40]. Later studies also found that trace sodium cyclamate can affect cell morphology, hinder cell movement, and even cause apoptosis [41]. In our experiments, we found that sodium cyclamate inhibited the proliferation, increased the apoptosis and necrosis in HRPECs. Besides, HRECs treated with sodium cyclamate affected tubule formation and migration in HRECs (Supplementary Files 5, 6). These results suggested a harmful effect of cyclamic acid on HRPECs and HRECs.

This study has some limitations. The first limitation is that the sample size of this study is small. The second limitation is that we did not sub-classify the samples according to the severity of the disease because of the limited sample size. There may be some differences between individuals with different degrees of disease which were ignored in this study. We need to pay more attention to collect samples in future studies. Third, although our study identified differential metabolites between wet AMD patients and normal people, as well as between different genotypes of *CFH* rs800292 and *HTRA1* rs10490924, the specific role of differential metabolites in the development of complex disease wAMD or its subtypes, still needs to be revealed by further investigation.

## MATERIALS AND METHODS

### Sample collection

This study was approved by the Ethics Committee of Sichuan Provincial People's Hospital (approval no. 2016(23)). Informed consents were obtained from all plasma donors. From 2016 to 2018, patients diagnosed with wet AMD (CNV patients and PCV patients) and participants without AMD were recruited from Sichuan Provincial People's Hospital of China. Other eye diseases (eye infections, diabetic retinopathy, etc.), diabetes, and people who have had any eye surgery were excluded. All participants underwent comprehensive eye examinations, including best-corrected vision assessment, fundus photography, optical coherence tomography (OCT)/optical coherence tomography angiography (OCTA), fluorescein angiogenesis (FA), or indocyanine green angiography (ICGA), which were used for wAMD diagnosis. Fasting plasma from 2 ml peripheral venous blood was collected from each participant and stored in a refrigerator at -80° C.

### LC-MS/MS analysis

Before chromatography-mass spectrometry analysis, sample extraction was performed. In short, the sample was taken out from the -80° C refrigerator, thawed, and vortexed for 10 seconds. Fifty microliters were placed into the EP tube, and 150 μL of precooled iced methanol (containing 1 μg/mL of 2-chlorophenylalanine as the internal standard) was added. Then, it was vortexed and centrifuged for 3 min at 12000 r/min. Then, the supernatant was centrifuged at 4° C for 10 min and absorbed into another new EP tube. The supernatant was centrifuged with 12000 r/min at 4° C for another 5 min. Finally, the supernatant was placed into the liner tube of the injection bottle for LC-MS/MS analysis. Chromatographic and mass spectrometry acquisition conditions and related data acquisition instrument systems are shown in Supplementary File 7.

### Data analysis

The demographic characteristics of the participants were described employing the mean and standard deviation. Metabolite differences between groups were compared by variance analysis and the chi-square test. Metabolomics data have the characteristics of "high dimension and mass", so it needs not only univariate statistical analysis but also multivariate statistical analysis.

Principal component analysis (PCA) is an unsupervised pattern recognition method for statistical analysis of multidimensional data and one of the commonly used dimensionality reduction techniques. It can derive a few principal components from the original variables and reveal the internal structure of multiple variables [61]. PCA and partial least squares discriminant analysis (PLS-DA) was used in multivariate statistical analysis. When using PCA to analyze the trend of separation between groups, we selected the first two and three features that best reflect the characteristics of data sets. PC1 represents the most obvious feature in the multidimensional data matrix, PC2 represents the most obvious feature in the data matrix other than PC1, and so on.

PLS-DA is a multivariate statistical analysis method with supervised pattern recognition that can maximize intergroup differentiation [62], which is conducive to finding differential metabolites. Based on the variable import in projection (VIP) of the PLS-DA model, we can combine the p-value or the fold change of univariate analysis to further screen the differential metabolites [63]. VIP value combined with p-value or fold change of univariate analysis was used to further screen differential metabolites. The screening criteria are as follows:

(1) Metabolites with fold change ≥ 2 and fold change ≤ 0.5 were selected. The difference in metabolites between the control group and the experimental group was more than 2 times or less than 0.5.

(2) Metabolites with a *p*-value < 0.05 were selected. The difference in metabolites in different groups was statistically significant.

(3) Metabolites with VIP ≥ 1 were selected. The VIP value indicates the influence intensity of the difference between the corresponding metabolites in the classification of samples in each group in the model. Generally speaking, the metabolites with VIP ≥ 1 are significantly different.

If the above three conditions were satisfied, the metabolite was significantly different between the groups.

### Public databases

The METLIN database (metlin.scripps.edu), MassBank database (http://www.massbank.jp/), HMDB database (http://www.hmdb.ca/), new drugs and metabolites mass spectrometry database (http://www.ualberta.ca/_gjones/mslid.htm), and the KEGG database (http://www.genome.jp/kegg/ligand.html) [64] were used for metabolite identification and pathway analysis.

### Genotyping of *CFH* rs80092 and *HTRA1* rs10490924

DNA was extracted from whole blood. The concentration of DNA was determined by using NanoDrop. Primers for CFH rs80092 (F:5' GATTGCAATGAACTTCCTCCA 3'; R:5' CCAGGCGATAGAGGGAGACT 3') and HTRA1 rs10490924 (F:5' TTGTGTGACGGGAAAAGACA3'; R:5' AAGCTTTGGGTTTCTGCTCA 3') were designed with Primer3 to PCR-amplify the 400–500bp region flanking the SNPs. The amplification was then Sanger sequenced on an Applied Biosystems (ABI) 3730 capillary sequencer. The Sanger sequencing results were analyzed with Sequencer software (ABI).

### Culture of HRPECs

HRPECs were obtained from ATCC (#CRL-2302). The cells were cultured in Dulbecco's modified Eagle's medium (Gibco, China) containing 10% fetal bovine serum (Gibco, Australia) and 1% penicillin-streptomycin (HyClone, USA). Cells were cultured in a CO_2_ incubator at 37° C and 5% CO_2_.

### Culture of HRECs

Primary human retinal endothelial cells were obtained from Cell Systems (#ACBRI 181). The cells were cultured in endothelial cell basal medium-2 (Lonza, USA) containing EGM^TM^-2 Single Quots Kit (Lonza, USA), 10% fetal bovine serum (Gibco, China), and 1% penicillin-streptomycin solution (HyClone, USA). The cells were cultured in a CO_2_ incubator at 37° C and 5% CO_2_.

### Chemicals

O-phosphorylethanolamine (Sigma, USA, #P0503-1G), hyodeoxycholic acid (Sigma, USA, H3878-5G), L-tryptophanamide (Selleck, USA, #S6155), cyclamic acid (Yuanye Biology, China, #S70017-5G), sodium cyclamate (Sigma, USA, #47827), curcumin [65] (Selleck, USA, #S1848), bevacizumab (Selleck, USA, #A2006). Blank control group (culture medium), negative control (0.1% DMSO group), curcumin (positive control group when studying the effect of differential metabolites on HRPECs), bevacizumab group (positive control group when studying the effect of differential metabolites on HRECs) were set.

### Detection of activity and proliferation inhibition of HRPECs

CCK-8 cell proliferation and cytotoxicity assay kit (Solarbio, China) was used according to the manufacturer's instructions. The HRPECs suspension was seeded in 96 well plates with 100 μL (about 1×10^4^ cells). After cells adhered to the wall, relevant reagents were added and incubated for 24 h and 48 h. Then, a 10 μL CCK-8 cell proliferation and cytotoxicity assay kit was added to each well, and cultured for 1-4 h. The optical density (OD) at 450 nm was determined by an enzyme-labeled instrument.

### Detection of apoptosis and necrosis of HRPECs

Hoechst 33342 / PI double stein kit (Solarbio, China) was used according to the manufacturer's instructions. The cell suspension was seeded on a 6-well plate with 2 ml cell suspension per well with about 10^6^ cells in each well. After the cells adhered to the wall, the culture medium was replaced with a culture medium containing related reagents and cultured for 24 h and 48 h. Then, the medium was discarded, cells were washed with phosphate-buffered saline (Gibco, China) once. Then, 1 ml of cell staining buffer, 5 uL of Hoechst staining solution, and 5 uL of PI staining solution were added and stained at 4° C for 20-30 minutes. After staining, phosphate-buffered saline was washed once and then observed under a fluorescence microscope.

### Tubule formation assay

First, growth factor reduced basement membrane matrix (Corning, USA) was spread on the 15 μ-slide angiogenesis (ibidi, Germany), 10 uL per well, 50 uL of HRECs suspension was added to each well (about 2×10^4^ cells per well), and cultured in a CO_2_ incubator at 37° C and 5% CO_2_ for 6 h, then observed under a microscope. The tube number was counted by image J software.

### Wound healing assay

The HRECs suspension (5×10^5^ cells/ml) was added into culture-insert (ibidi, Germany) with 10 uL per well. They were cultured in a CO_2_ incubator at 37° C with 5% CO_2_. After cell adhesion, the medium was replaced with corresponding new medium containing reagents and observed at 6h, 12h, and 24h respectively.

### Statistical analysis

Analysis 1.6.3, MWDB, multi quart software, R, KEGG database were used for qualitative and quantitative analysis of metabolites. Graphpad prism was used to analyze the results of *in vitro* experiments, and an independent t-test was used to analyze the significant differences.

## Supplementary Material

Supplementary File 1

Supplementary File 2

Supplementary File 3

Supplementary File 4

Supplementary File 5

Supplementary File 6

Supplementary File 7
