# Supplementary material for: Untargeted metabolomics for uncovering plasma biological markers of wet age-related macular degeneration
Source: Aging (Albany NY). 2021 May 4;13(10):13968–4000. doi: 10.18632/aging.203006 (PMC8202859; doi:10.18632/aging.203006)
Supplement: Supplementary File 1 [file aging-13-203006-s001.pdf]

SUPPLEMENTARY FILE

Supplementary File 1. Sample collections for PCV and CNV cases and controls.

Disease	Number		Age (mean ± s.d., in years, range)		Percent female (%)	
	Cases	Controls	Cases	Controls	Cases	Controls
wAMD	127	50	71.1 ± 8.4 (43.0-94.0)	68.5±9.0 (50.0–87.0)	39	39
CNV	60	50	74.9 ± 7.8 (50.0-94.0)	68.5±9.0 (50.0–87.0)	45	39
PCV	67	50	67.7 ± 9.0 (43.0–90.0)	68.5±9.0 (50.0–87.0)	33	39
