# Supplementary material for: Untargeted metabolomics for uncovering plasma biological markers of wet age-related macular degeneration
Source: Aging (Albany NY). 2021 May 4;13(10):13968–4000. doi: 10.18632/aging.203006 (PMC8202859; doi:10.18632/aging.203006)
Supplement: Supplementary File 4 [file aging-13-203006-s004.docx]

**Supplementary File 4. The effects of cyclamic acid on activity and proliferation of HRPECs.**

|  | **Group** | **Mean±SEM (%)** | **Comparing group *P* value** | |
| --- | --- | --- | --- | --- |
| Cell activity (24h) | \| Con \| \| --- \| \| DMSO \| \| Cur \| \| C-10 \| \| C-20 \| \| C-30 \| \| C-40 \| | \| 1.064 ± 0.034 \| \| --- \| \| 0.996 ± 0.037 \| \| 0.740 ± 0.048 \| \| 1.055 ± 0.042 \| \| 1.020 ± 0.024 \| \| 0.479 ± 0.020 \| \| 0.214 ± 0.004 \| | \| Con vs DMSO \| \| --- \| \| DMSO vs Cur \| \| C-10 vs Con \| \| C-20 vs Con \| \| C-30 vs Con \| \| C-40 vs Con \| \| C-10 vs Cur \| \| C-20 vs Cur \| \| C-30 vs Cur \| \| C-40 vs Cur \| | \| 0.222 \| \| --- \| \| 0.006 \| \| 0.873 \| \| 0.326 \| \| 0.000 \| \| 0.000 \| \| 0.003 \| \| 0.002 \| \| 0.002 \| \| 0.000 \| |
| Cell activity (48h) | \| Con \| \| --- \| \| DMSO \| \| Cur \| \| C-10 \| \| C-20 \| \| C-30 \| \| C-40 \| | \| 1.611 ± 0.022 \| \| --- \| \| 1.534 ± 0.030 \| \| 0.863 ± 0.031 \| \| 1.433 ± 0.028 \| \| 1.143 ± 0.043 \| \| 0.356 ± 0.023 \| \| 0.225 ± 0.003 \| | \| Con vs DMSO \| \| --- \| \| DMSO vs Cur \| \| C-10 vs Con \| \| C-20 vs Con \| \| C-30 vs Con \| \| C-40 vs Con \| \| C-10 vs Cur \| \| C-20 vs Cur \| \| C-30 vs Cur \| \| C-40 vs Cur \| | \| 0.084 \| \| --- \| \| 0.000 \| \| 0.003 \| \| 0.000 \| \| 0.000 \| \| 0.000 \| \| 0.000 \| \| 0.002 \| \| 0.000 \| \| 0.000 \| |
| Inhibition rate (24h) | \| Cur \| \| --- \| \| C-10 \| \| C-20 \| \| C-30 \| \| C-40 \| | \| 16.335 ± 0.499 \| \| --- \| \| 11.529 ± 0.247 \| \| 16.591 ± 0.708 \| \| 21.329 ± 0.870 \| \| 28.750 ± 1.249 \| | \| C-10 vs Cur \| \| --- \| \| C-20 vs Cur \| \| C-30 vs Cur \| \| C-40 vs Cur \| | \| 0.001 \| \| --- \| \| 0.783 \| \| 0.008 \| \| 0.001 \| |
| Inhibition rate (48h) | \| Cur \| \| --- \| \| C-10 \| \| C-20 \| \| C-30 \| \| C-40 \| | \| 17.331 ± 0.543 \| \| --- \| \| 13.468 ± 0.252 \| \| 21.462 ± 0.485 \| \| 22.061 ± 1.053 \| \| 33.937 ± 0.356 \| | \| C-10 vs Cur \| \| --- \| \| C-20 vs Cur \| \| C-30 vs Cur \| \| C-40 vs Cur \| | \| 0.003 \| \| --- \| \| 0.005 \| \| 0.016 \| \| 0.000 \| |

|  | **Group** | **Mean±SEM (%)** | **Comparing group *P* value** | |
| --- | --- | --- | --- | --- |
| Cell activity (24h) | \| Con \| \| --- \| \| DMSO \| \| Cur \| \| H-40 \| \| H-80 \| \| H-120 \| \| H-160 \| | \| 0.819 ± 0.038 \| \| --- \| \| 0.788 ± 0.021 \| \| 0.596 ± 0.026 \| \| 0.813 ± 0.043 \| \| 0.754 ± 0.025 \| \| 0.736 ± 0.013 \| \| 0.735 ± 0.022 \| | \| Con vs DMSO \| \| --- \| \| DMSO vs Cur \| \| H-40 vs DMSO \| \| H-80 vs DMSO \| \| H-120 vs DMSO \| \| H-160 vs DMSO \| \| H-40 vs Cur \| \| H-80 vs Cur \| \| H-120 vs Cur \| \| H-160 vs Cur \| | \| 0.503 \| \| --- \| \| 0.001 \| \| 0.925 \| \| 0.203 \| \| 0.085 \| \| 0.105 \| \| 0.005 \| \| 0.004 \| \| 0.003 \| \| 0.006 \| |
| Cell activity (48h) | \| Con \| \| --- \| \| DMSO \| \| Cur \| \| H-40 \| \| H-80 \| \| H-120 \| \| H-160 \| | \| 1.938 ± 0.016 \| \| --- \| \| 1.930 ± 0.059 \| \| 1.189 ± 0.122 \| \| 1.984 ± 0.091 \| \| 1.935 ± 0.021 \| \| 1.913 ± 0.100 \| \| 1.900 ± 0.071 \| | \| Con vs DMSO \| \| --- \| \| DMSO vs Cur \| \| H-40 vs DMSO \| \| H-80 vs DMSO \| \| H-120 vs DMSO \| \| H-160 vs DMSO \| \| H-40 vs Cur \| \| H-80 vs Cur \| \| H-120 vs Cur \| \| H-160 vs Cur \| | \| 0.900 \| \| --- \| \| 0.002 \| \| 0.638 \| \| 0.907 \| \| 0.287 \| \| 0.622 \| \| 0.002 \| \| 0.001 \| \| 0.004 \| \| 0.002 \| |
| Inhibition rate (24h) | \| Cur \| \| --- \| \| H-40 \| \| H-80 \| \| H-120 \| \| H-160 \| | \| 13.002 ± 2.274 \| \| --- \| \| 8.122 ± 1.286 \| \| 8.796 ± 1.238 \| \| 11.714 ± 2.519 \| \| 13.074 ± 0.865 \| | \| H-40 vs Cur \| \| --- \| \| H-80 vs Cur \| \| H-120 vs Cur \| \| H-160 vs Cur \| | \| 0.135 \| \| --- \| \| 0.180 \| \| 0.724 \| \| 0.978 \| |
| Inhibition rate (48h) | \| Cur \| \| --- \| \| H-40 \| \| H-80 \| \| H-120 \| \| H-160 \| | \| 14.649 ± 0.820 \| \| --- \| \| 9.319 ± 1.254 \| \| 11.997 ± 2.306 \| \| 13.426 ± 1.153 \| \| 15.513 ± 0.169 \| | \| H-40 vs Cur \| \| --- \| \| H-80 vs Cur \| \| H-120 vs Cur \| \| H-160 vs Cur \| | \| 0.024 \| \| --- \| \| 0.210 \| \| 0.436 \| \| 0.361 \| |

The effects of hyodeoxycholic acid on activity and proliferation of hRPE cells

|  | **Group** | **Mean±SEM (%)** | **Comparing group *P* value** | |
| --- | --- | --- | --- | --- |
| Cell activity (24h) | \| Con \| \| --- \| \| DMSO \| \| Cur \| \| L-25 \| \| L-50 \| \| L-75 \| \| L-100 \| | \| 0.896 ± 0.018 \| \| --- \| \| 0.840 ± 0.030 \| \| 0.676 ± 0.012 \| \| 0.873 ± 0.011 \| \| 0.857 ± 0.038 \| \| 0.856 ± 0.028 \| \| 0.854 ± 0.027 \| | \| Con vs DMSO \| \| --- \| \| DMSO vs Cur \| \| L-25 vs DMSO \| \| L-50 vs DMSO \| \| L-75 vs DMSO \| \| L-100 vs DMSO \| \| L-25 vs Cur \| \| L-50 vs Cur \| \| L-75 vs Cur \| \| L-100 vs Cur \| | \| 0.542 \| \| --- \| \| 0.002 \| \| 0.391 \| \| 0.276 \| \| 0.305 \| \| 0.250 \| \| 0.000 \| \| 0.004 \| \| 0.001 \| \| 0.001 \| |
| Cell activity (48h) | \| Con \| \| --- \| \| DMSO \| \| Cur \| \| L-25 \| \| L-50 \| \| L-75 \| \| L-100 \| | \| 1.507 ± 0.017 \| \| --- \| \| 1.515 ± 0.028 \| \| 0.972 ± 0.023 \| \| 1.518 ± 0.016 \| \| 1.497 ± 0.023 \| \| 1.490 ± 0.016 \| \| 1.475 ± 0.070 \| | \| Con vs DMSO \| \| --- \| \| DMSO vs Cur \| \| L-25 vs DMSO \| \| L-50 vs DMSO \| \| L-75 vs DMSO \| \| L-100 vs DMSO \| \| L-25 vs Cur \| \| L-50 vs Cur \| \| L-75 vs Cur \| \| L-100 vs Cur \| | \| 0.821 \| \| --- \| \| 0.000 \| \| 0.644 \| \| 0.721 \| \| 0.495 \| \| 0.666 \| \| 0.000 \| \| 0.000 \| \| 0.000 \| \| 0.001 \| |
| Inhibition rate (24h) | \| Cur \| \| --- \| \| L-25 \| \| L-50 \| \| L-75 \| \| L-100 \| | \| 13.546 ± 0.65 \| \| --- \| \| 12.026 ± 0.403 \| \| 13.121 ± 0.604 \| \| 15.364 ± 0.391 \| \| 16.289 ± 1.079 \| | \| L-25 vs Cur \| \| --- \| \| L-50 vs Cur \| \| L-75 vs Cur \| \| L-100 vs Cur \| | \| 0.105 \| \| --- \| \| 0.646 \| \| 0.065 \| \| 0.091 \| |
| Inhibition rate (48h) | \| Cur \| \| --- \| \| L-25 \| \| L-50 \| \| L-75 \| \| L-100 \| | \| 13.863 ± 0.485 \| \| --- \| \| 14.707 ± 2.193 \| \| 16.255 ± 0.988 \| \| 18.298 ± 1.362 \| \| 20.432 ± 1.841 \| | \| L-25 vs Cur \| \| --- \| \| L-50 vs Cur \| \| L-75 vs Cur \| \| L-100 vs Cur \| | \| 0.726 \| \| --- \| \| 0.095 \| \| 0.037 \| \| 0.026 \| |

The effects of L-tryptophanamide on activity and proliferation of HRPECs

The effects of O-Phosphorylethanolamine on activity and proliferation of HRPECs

|  | **Group** | **Mean±SEM (%)** | **Comparing group *P* value** | |
| --- | --- | --- | --- | --- |
| Cell activity (24h) | \| Con \| \| --- \| \| DMSO \| \| Cur \| \| O-5 \| \| O-10 \| \| O-15 \| \| O-20 \| | \| 0.958 ± 0.018 \| \| --- \| \| 1.002 ± 0.033 \| \| 0.742 ± 0.019 \| \| 1.051 ± 0.018 \| \| 1.058 ± 0.035 \| \| 1.064 ± 0.011 \| \| 1.109 ± 0.026 \| | \| Con vs DMSO \| \| --- \| \| DMSO vs Cur \| \| O-5 vs Con \| \| O-10 vs Con \| \| O-15 vs Con \| \| O-20 vs Con \| \| O-5 vs Cur \| \| O-10 vs Cur \| \| O-15 vs Cur \| \| O-20 vs Cur \| | \| 0.286 \| \| --- \| \| 0.001 \| \| 0.010 \| \| 0.045 \| \| 0.002 \| \| 0.003 \| \| 0.000 \| \| 0.000 \| \| 0.000 \| \| 0.000 \| |
| Cell activity (48h) | \| Con \|  \| \| --- \| --- \| \| DMSO \|  \| \| Cur \|  \| \| O-5 \|  \| \| O-10 \|  \| \| O-15 \|  \| \| O-20 \|  \| | \| 1.713 ± 0.037 \| \| --- \| \| 1.627 ± 0.074 \| \| 0.907 ± 0.035 \| \| 1.940 ± 0.049 \| \| 1.953 ± 0.053 \| \| 2.182 ± 0.089 \| \| 2.216 ± 0.027 \| | \| Con vs DMSO \| \| --- \| \| DMSO vs Cur \| \| 5 vs Con \| \| 10 vs Con \| \| 15 vs Con \| \| 20 vs Con \| \| O-5 vs Cur \| \| O-10 vs Cur \| \| O-15 vs Cur \| \| O-20 vs Cur \| | \| 0.338 \| \| --- \| \| 0.000 \| \| 0.010 \| \| 0.010 \| \| 0.003 \| \| 0.000 \| \| 0.000 \| \| 0.000 \| \| 0.000 \| \| 0.000 \| |
| Inhibition rate (24h) | \| Cur \| \| --- \| \| O-5 \| \| O-10 \| \| O-15 \| \| O-20 \| | \| 14.380 ± 0.738 \| \| --- \| \| -0.361 ± 0.677 \| \| -2.317 ± 1.410 \| \| -2.493 ± 1.417 \| \| -4.005 ± 0.670 \| | \| O-5 vs Cur \| \| --- \| \| O-10 vs Cur \| \| O-15 vs Cur \| \| O-20 vs Cur \| | \| 0.000 \| \| --- \| \| 0.001 \| \| 0.001 \| \| 0.000 \| |
| Inhibition rate (48h) | \| Cur \| \| --- \| \| O-5 \| \| O-10 \| \| O-15 \| \| O-20 \| | \| 15.895 ± 0.055 \| \| --- \| \| -1.793 ± 0.454 \| \| -5.817 ± 0.510 \| \| -7.855 ± 0.350 \| \| -8.743 ± 0.078 \| | \| O-5 vs Cur \| \| --- \| \| O-10 vs Cur \| \| O-15 vs Cur \| \| O-20 vs Cur \| | \| 0.000 \| \| --- \| \| 0.000 \| \| 0.000 \| \| 0.000 \| |
