# Supplementary material for: Untargeted metabolomics for uncovering plasma biological markers of wet age-related macular degeneration
Source: Aging (Albany NY). 2021 May 4;13(10):13968–4000. doi: 10.18632/aging.203006 (PMC8202859; doi:10.18632/aging.203006)
Supplement: Supplementary File 5 [file aging-13-203006-s005.pdf]

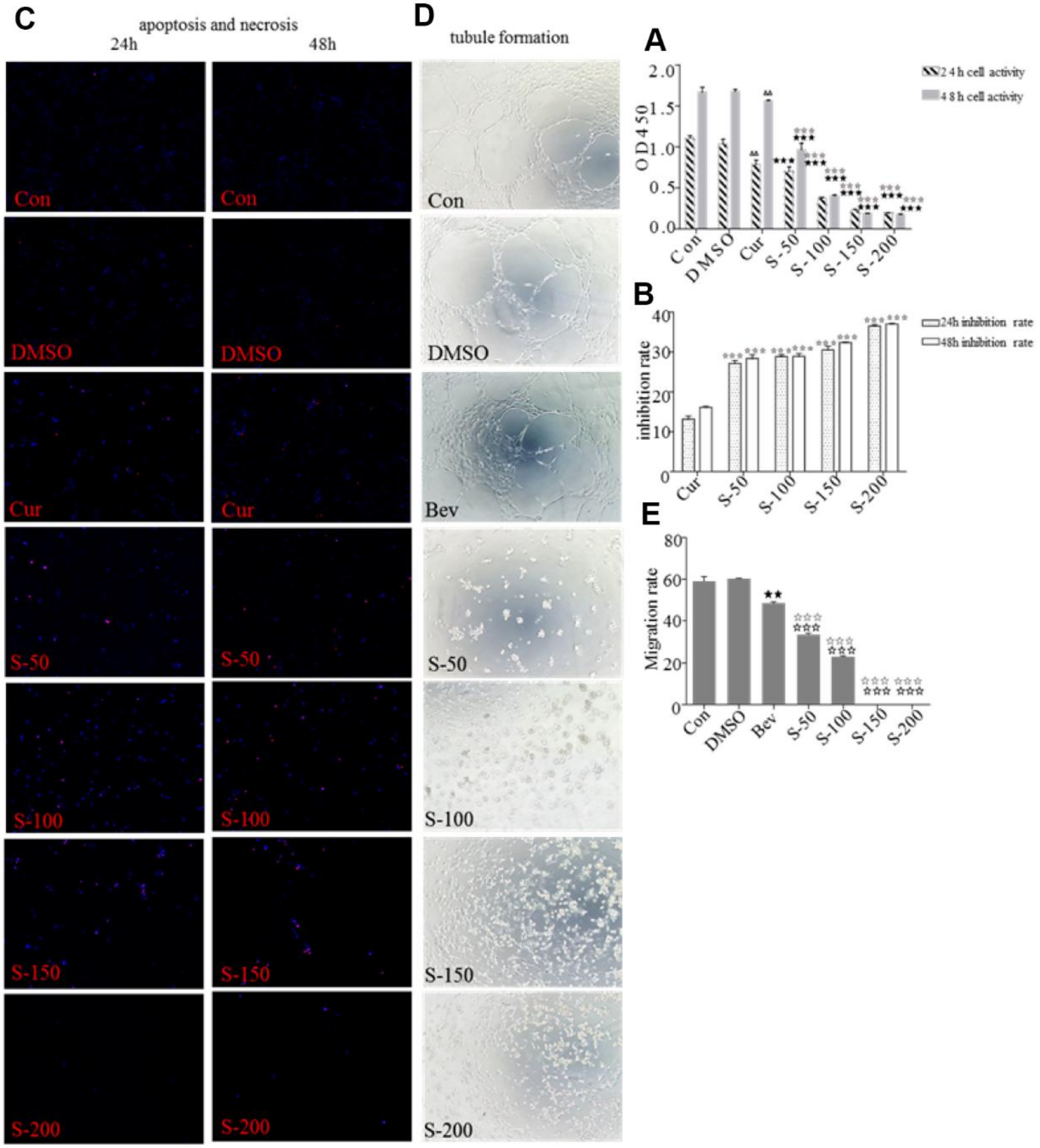


**Supplementary File 5. The effects of sodium cyclamate on HRPECs and HRECs.** The effects of sodium cyclamate on HRPECs and HRECs. (**A**, **B**) show the effect of sodium cyclamate on the activity and proliferation of HRPECs, (**C**) shows the effect of sodium cyclamate on the proliferation and apoptosis of HRPECs, and (**D**) and e show the effect of sodium cyclamate on the tubule formation and migration of HRECs. S-50, S-100, s-150 and S-200 represent 50 µmol/ml, 100 µmol/ml, 150 µmol/ml and 200 µmol/ml sodium cyclamate, respectively. (**A**, **B**), ΔΔ and ☆☆ ΔΔΔ indicated that the positive control group (cur) was significantly different from the 0.1% DMSO group (0.001<*P*<0.01, *P*<0.001). ☆, and ☆☆☆ respectively indicated that there were significant differences between this group and the positive control group, and the corresponding values were 0.01<*P*<0.05, 0.001<*P*<0.01, *P*<0.001. ★,★★ and ★★★ respectively indicated that there were significant differences between this group and the blank control group (con), and the corresponding values were 0.01<*P*<0.05, 0.001<*P*<0.01, *P*<0.001. In (**E**), The black solid Pentagram indicates that there is a difference between bevacizumab (Bev) group and the control (Con) group, 0.001<*P*<0.01. The five-pointed stars with a gray border, black border, and gray solid border indicate that there are significant differences between the group and Bev group, con group, and DMSO group, respectively. The number of five-pointed stars 1, 2 and 3 represent the current 0.01 < *P*< 0.05, 0.001 < *P* < 0.01 and *P* < 0.001, respectively.
