# Supplementary material for: Untargeted metabolomics for uncovering plasma biological markers of wet age-related macular degeneration
Source: Aging (Albany NY). 2021 May 4;13(10):13968–4000. doi: 10.18632/aging.203006 (PMC8202859; doi:10.18632/aging.203006)
Supplement: Supplementary File 6 [file aging-13-203006-s006.docx]

**Supplementary File 6. The effects of sodium cyclamate on the activity and cytotoxicity of HRPECs.**

|  | **Group** | **Mean±SEM (%)** | **Comparing group  *P* value** | |
| --- | --- | --- | --- | --- |
| Cell activity (24h) | \| Con \| \| --- \| \| DMSO \| \| Cur \| \| S-50 \| \| S-100 \| \| S-150 \| \| S-200 \| | \| 1.107 ± 0.016 \| \| --- \| \| 1.040 ± 0.027 \| \| 0.787 ± 0.026 \| \| 0.702 ± 0.027 \| \| 0.379 ± 0.003 \| \| 0.228 ± 0.007 \| \| 0.196 ± 0.003 \| | \| Con vs DMSO \| \| --- \| \| DMSO vs Cur \| \| S-50 vs Con \| \| S-100 vs Con \| \| S-150 vs Con \| \| S-200 vs Con \| \| S-50 vs Cur \| \| S-100 vs Cur \| \| S-150 vs Cur \| \| S-200 vs Cur \| | \| 0.077 \| \| --- \| \| 0.006 \| \| 0.000 \| \| 0.000 \| \| 0.000 \| \| 0.000 \| \| 0.065 \| \| 0.000 \| \| 0.000 \| \| 0.000 \| |
| Cell activity (48h) | \| Con \| \| --- \| \| DMSO \| \| Cur \| \| S-50 \| \| S-100 \| \| S-150 \| \| S-200 \| | \| 1.669 ± 0.061 \| \| --- \| \| 1.680 ± 0.026 \| \| 1.563 ± 0.009 \| \| 0.969 ± 0.077 \| \| 0.404 ± 0.011 \| \| 0.190 ± 0.002 \| \| 0.178 ± 0.002 \| | \| Con vs DMSO \| \| --- \| \| DMSO vs Cur \| \| S-50 vs Con \| \| S-100 vs Con \| \| S-150 vs Con \| \| S-200 vs Con \| \| S-50 vs Cur \| \| S-100 vs Cur \| \| S-150 vs Cur \| \| S-200 vs Cur \| | \| 0.876 \| \| --- \| \| 0.006 \| \| 0.000 \| \| 0.000 \| \| 0.000 \| \| 0.000 \| \| 0.000 \| \| 0.000 \| \| 0.000 \| \| 0.000 \| |
| Inhibition rate (24h) | \| Cur \| \| --- \| \| S-50 \| \| S-100 \| \| S-150 \| \| S-200 \| | \| 13.229 ± 0.829 \| \| --- \| \| 27.172 ± 0.780 \| \| 28.919 ± 0.540 \| \| 30.581 ± 0.910 \| \| 36.554 ± 0.419 \| | \| S-50 vs Cur \| \| --- \| \| S-100 vs Cur \| \| S-150 vs Cur \| \| S-200 vs Cur \| | \| 0.000 \| \| --- \| \| 0.000 \| \| 0.000 \| \| 0.000 \| |
| Inhibition rate (48h) | \| Cur \| \| --- \| \| S-50 \| \| S-100 \| \| S-150 \| \| S-200 \| | \| 16.174 ± 0.310 \| \| --- \| \| 28.413 ± 0.975 \| \| 28.934 ± 0.764 \| \| 32.410 ± 0.153 \| \| 37.059 ± 0.216 \| | \| S-50 vs Cur \| \| --- \| \| S-100 vs Cur \| \| S-150 vs Cur \| \| S-200 vs Cur \| | \| 0.000 \| \| --- \| \| 0.000 \| \| 0.000 \| \| 0.000 \| |

The effects of sodium cyclamate on apoptosis and necrosis of HRPECs

|  | **Group** | **Mean±SEM (%)** | **Comparing group  *P* value** | |
| --- | --- | --- | --- | --- |
| Apoptosis rate (24h) | \| Con \| \| --- \| \| DMSO \| \| Cur \| \| S-50 \| \| S-100 \| \| S-150 \| \| S-200 \| | \| 3.863 ± 0.669 \| \| --- \| \| 4.414 ± 0.400 \| \| 11.720 ± 0.165 \| \| 4.271 ± 0.296 \| \| 9.725 ± 0.580 \| \| 29.950 ± 0.968 \| \| 32.840 ± 6.615 \| | \| Con vs DMSO \| \| --- \| \| DMSO vs Cur \| \| S-50 vs Con \| \| S-100 vs Con \| \| S-150 vs Con \| \| S-200 vs Con \| \| S-50 vs Cur \| \| S-100 vs Cur \| \| S-150 vs Cur \| \| S-200 vs Cur \| | \| 0.518 \| \| --- \| \| 0.000 \| \| 0.607 \| \| 0.003 \| \| 0.000 \| \| 0.012 \| \| 0.000 \| \| 0.030 \| \| 0.000 \| \| 0.013 \| |
| Apoptosis rate (48h) | \| Con \| \| --- \| \| DMSO \| \| Cur \| \| S-50 \| \| S-100 \| \| S-150 \| \| S-200 \| | \| 6.449 ± 0.370 \| \| --- \| \| 7.611 ± 0.315 \| \| 13.22 ± 0.763 \| \| 5.187 ± 0.552 \| \| 12.180 ± 1.173 \| \| 31.550 ± 4.388 \| \| 35.850 ± 0.263 \| | \| Con vs DMSO \| \| --- \| \| DMSO vs Cur \| \| S-50 vs Con \| \| S-100 vs Con \| \| S-150 vs Con \| \| S-200 vs Con \| \| S-50 vs Cur \| \| S-100 vs Cur \| \| S-150 vs Cur \| \| S-200 vs Cur \| | \| 0.227 \| \| --- \| \| 0.009 \| \| 0.015 \| \| 0.000 \| \| 0.002 \| \| 0.008 \| \| 0.001 \| \| 0.008 \| \| 0.009 \| \| 0.003 \| |
| Necrosis rate (24h) | \| Con \| \| --- \| \| DMSO \| \| Cur \| \| S-50 \| \| S-100 \| \| S-150 \| \| S-200 \| | \| 0.127 ± 0.001 \| \| --- \| \| 0.127 ± 0.001 \| \| 1.411 ± 0.243 \| \| 1.413 ± 0.311 \| \| 2.962 ± 0.201 \| \| 24.850 ± 3.528 \| \| 25.310 ± 5.083 \| | \| Con vs DMSO \| \| --- \| \| DMSO vs Cur \| \| S-50 vs Con \| \| S-100 vs Con \| \| S-150 vs Con \| \| S-200 vs Con \| \| S-50 vs Cur \| \| S-100 vs Cur \| \| S-150 vs Cur \| \| S-200 vs Cur \| | \| 0.075 \| \| --- \| \| 0.003 \| \| 0.130 \| \| 0.010 \| \| 0.005 \| \| 0.000 \| \| 0.001 \| \| 0.497 \| \| 0.015 \| \| 0.000 \| |
| Necrosis rate (48h) | \| Con \| \| --- \| \| DMSO \| \| Cur \| \| S-50 \| \| S-100 \| \| S-150 \| \| S-200 \| | \| 0.133 ± 0.025 \| \| --- \| \| 0.133 ± 0.025 \| \| 4.167 ± 1.381 \| \| 1.675 ± 0.159 \| \| 3.485 ± 1.101 \| \| 26.510 ± 4.733 \| \| 27.010 ± 4.354 \| | \| Con vs DMSO \| \| --- \| \| DMSO vs Cur \| \| S-50 vs Con \| \| S-100 vs Con \| \| S-150 vs Con \| \| S-200 vs Con \| \| S-50 vs Cur \| \| S-100 vs Cur \| \| S-150 vs Cur \| \| S-200 vs Cur \| | \| 0.154 \| \| --- \| \| 0.043 \| \| 0.001 \| \| 0.039 \| \| 0.005 \| \| 0.004 \| \| 0.148 \| \| 0.719 \| \| 0.011 \| \| 0.008 \| |

| **Group** | **Mean±SEM** | **Comparing group  *P* value** | |
| --- | --- | --- | --- |
| \| Con \| \| --- \| \| DMSO \| \| Bev \| \| S-50 \| \| S-100 \| \| S-150 \| \| S-200 \| | \| 59.161 ± 2.126 \| \| --- \| \| 60.348 ± 0.180 \| \| 48.624 ± 0.637 \| \| 33.697 ± 0.564 \| \| 23.102 ± 0.361 \| \| 0.000 ± 0.000 \| \| 0.000 ± 0.000 \| | \| Con vs DMSO \| \| --- \| \| Con vs Bev \| \| S-50 vs Con \| \| S-100 vs Con \| \| S-150 vs Con \| \| S-200 vs Con \| \| S-50 vs Bev \| \| S-100 vs Bev \| \| S-150 vs Bev \| \| S-200 vs Bev \| | \| 0.608 \| \| --- \| \| 0.009 \| \| 0.000 \| \| 0.000 \| \| 0.000 \| \| 0.000 \| \| 0.000 \| \| 0.000 \| \| 0.000 \| \| 0.000 \| |

The effects of sodium cyclamate on migration rate of HRECs
