# Supplementary material for: Untargeted metabolomics for uncovering plasma biological markers of wet age-related macular degeneration
Source: Aging (Albany NY). 2021 May 4;13(10):13968–4000. doi: 10.18632/aging.203006 (PMC8202859; doi:10.18632/aging.203006)
Supplement: Supplementary File 7 [file aging-13-203006-s007.pdf]

Supplementary File 7

The condition of LC-MS

Data acquisition instrument system mainly includes Ultra Performance Liquid Chromatography (UPLC) (Shim-pack UFLC SHIMADZU CBM30A, https://www.shimadzu.com/) and Tandem mass spectrometry (MS / MS) (QTRAP ®, https://sciex.com/).

The liquid phase conditions mainly include:

1) Chromatographic column: Waters ACQUITY UPLC HSS T3 C18 1.8 µm, 2.1 mm*100 mm;

2) Mobile phase: A phase is ultrapure water (0.04% acetic acid), B phase is acetonitrile (0.04% acetic acid);

3) Elution gradient: 0 min water / acetonitrile (95:5 V / V), 11.0 min 5:95 V / V, 12.0 min 5:95 V / V, 12.1 min 95:5 V / V, 14.0 min 95:5 V / V;

4) Flow rate 0.4 ml / min; column temperature 40° C; injection volume 2 µL.

Mass spectrometry conditions mainly include:

The electrospray ionization (ESI) temperature is 500 degrees, the mass spectrum voltage is 5500 V (positive), -4500 V (negative), the ion source gas I (GS I) 55 GS, the gas is (60), the air curtain gas (25), the collision induced ionization is high. In the triple quadrupole (qtrap), each ion pair is scanned and detected according to the optimized de clustering potential (DP) and collision energy (CE) [1].

REFERENCES

1. Chen W, Gong L, Guo Z, Wang W, Zhang H, Liu X, Yu S, Xiong L, Luo J. A novel integrated method for large-scale detection, identification, and quantification of widely targeted metabolites: application in the study of rice metabolomics. Mol Plant. 2013; 6:1769–80. https://doi.org/10.1093/mp/sst080 PMID:23702596
